# Stoneflies (Insecta, Plecoptera) of the Midwest, USA: species checklist derived from nearly 170 Years of collection records

**DOI:** 10.3897/BDJ.14.e208358

**Published:** 2026-09-02

**Authors:** Phillip N. Hogan, Ralph Edward DeWalt, Scott A. Grubbs

**Affiliations:** 1 University of Illinois, Urbana-Champaign, Urbana, United States of America University of Illinois, Urbana-Champaign Urbana United States of America; 2 University of Illinois, Illinois Natural History Survey, Champaign, United States of America University of Illinois, Illinois Natural History Survey Champaign United States of America; 3 Western Kentucky University, Bowling Green, United States of America Western Kentucky University Bowling Green United States of America

**Keywords:** Faunisitics, Landscape, Natural History Museums, Species Distributions

## Abstract

**Background:**

The landscape in the Midwest, USA (limited to the states of Illinois, Indiana, Iowa, Michigan, Minnesota, Ohio and Wisconsin) has undergone dramatic shifts from the historic natural settings to agricultural and urbanised landscapes over the past two centuries following European colonisation. These shifts have restructured community assemblages and species distributions of aquatic insects, particularly stoneflies, an insect order susceptible to regional and local extirpations following anthropogenic, climatic and environmental alterations. Within the Midwest, species extirpations at a broad scale have been documented for multiple states, including Illinois, Indiana, Michigan and Ohio. Illinois and Minnesota appear to have lost endemics to extinction. To better understand how species distributions have shifted over the past two centuries, we have compiled records of species occurrences from the mid-19^th^ century to the present day.

**New information:**

In preparation for a comprehensive assessment of the Midwest stonefly fauna, we present a species occurrence dataset composed of approximately 50,000 records. This dataset spans from the early 1860s through 2025. These data have been compiled from 26 regional natural history museums, a review of published literature records and collecting efforts by the authors since 1987. This dataset presents occurrence records for 157 regional species from 41 genera, representing nine of ten Nearctic stonefly families. Of these species, 21 are new state records, including three species recorded for the first time within the Midwest region. The new state records include two from Illinois, two from Indiana, one from Iowa, one from Michigan, nine from Minnesota, five from Ohio and seven from Wisconsin. Overall, these updates bring the total known richness for Illinois to 85, Indiana to 93, Iowa to 47, Michigan to 71, Minnesota to 65, Ohio to 109 and Wisconsin to 68. State richness declines along east-west and south-north gradients. The five most species-rich families were Perlidae (n = 42), Perlodidae (n = 38), Capniidae (n = 21), Chloroperlidae (n = 15) and Nemouridae (n = 15). The contemporary Midwest fauna is diminished compared to historical community richness and composition, through both regional and state extirpations and extinctions. Despite intensive contemporary collection efforts throughout the region, 13 species are known solely from historical collections. Each Midwest state experienced extirpations. The greatest losses occurred in Illinois, with 16 species that were absent in collections made after 1970. This was followed by Indiana (n = 9), Michigan (n = 8), Ohio (n = 8), Minnesota (n = 7), Wisconsin (n = 2) and Iowa (n = 1). The greatest losses were within the families Perlidae and Perlodidae, with 17 and eight species extirpated from at least one state by 1970, respectively.

## Introduction

Stoneflies (Insecta, Plecoptera) are a small order of aquatic insects that are most species-rich in temperate, mountain streams (Fochetti and Tierno de Figueroa 2007, DeWalt and Ower 2019). Approximately 4,300 extant species of stoneflies are currently described, with 17% of the global diversity found within the Nearctic realm (DeWalt and Ower 2019, DeWalt et al. 2026). Amongst the most sensitive insects to changes in water quality, stoneflies are often utilised in biomonitoring studies (Hilsenhoff 1977, Lenat 1993). This sensitivity is, in part, due to the wide variety of life history strategies employed, feeding mechanisms present and ecological requirements of stoneflies (Eichert et al. 2025). Stoneflies are amongst the first aquatic insects extirpated due to landscape disturbances and declining water quality (Pond 2011).

The Midwest, USA has a rich taxonomic history with investigations into the stonefly fauna spanning over two centuries. Efforts to document and describe the Midwest fauna began in the early 19^th^ century with the description of several species (Say 1823). Taxonomic and faunistic efforts continued throughout the 20^th^ and 21^st^ centuries, with many specimens archived in regional natural history museums (Newman et al. 2021). Structured upon these archived specimens, contemporary efforts focused on documenting faunal changes that occurred within the states of Illinois (DeWalt et al. 2005), Indiana (Newman et al. 2021), Michigan (Grubbs et al. 2012) and Ohio (DeWalt et al. 2012, DeWalt et al. 2016).

Primary biodiversity studies derived from reviewed and taxonomically updated species occurrence datasets have become increasingly important in the face of rapidly changing climates, anthropogenically induced landscape modifications and the introduction of non-native species. The resulting occurrence data provide valuable insights into the spatial and temporal components of the geographic range for a species (Elith and Leathwick 2009). The primary objective of this manuscript is to present a comprehensive, publicly available stonefly species occurrence dataset for the Midwest. These data document distributional, phenological and community shifts, and conservation status within the region. This effort follows that of Hart et al. (2025) for Arkansas, Myers et al. (2025) for New York and Cook et al. (2026) for West Virginia.

### History of stonefly studies in the Midwest, USA region

Stoneflies have been studied for a long time in the Midwest with the first published work dating back to Say (1823). From the Ohio River near Cincinnati, he described *Alloperla imbecilla* (Say, 1823) and *Isoperla bilineata* (Say, 1823) (Table 1). Benjamin D. Walsh, Illinois' first state entomologist, was an early contributor to Midwest stoneflies, describing nine new species and compiling a list of 17 species from the vicinity of Rock Island, Illinois (Walsh 1862). Five of his species were later placed in synonymy, leaving four of his described species valid today: *Agnetina flavescens* (Walsh, 1862), *Perlesta decipiens* (Walsh, 1862), *Isoperla nana* (Walsh, 1862) and *Isogenoides varians* (Walsh, 1862). Of the 17 species listed by Walsh (1862), 13 are currently valid when accounting for taxonomic updates and excluding a nomen dubium. Following Walsh's work, tallies of the number of species known from each Midwest state could be extracted from Needham and Claassen (1925).

**Figure 1.**
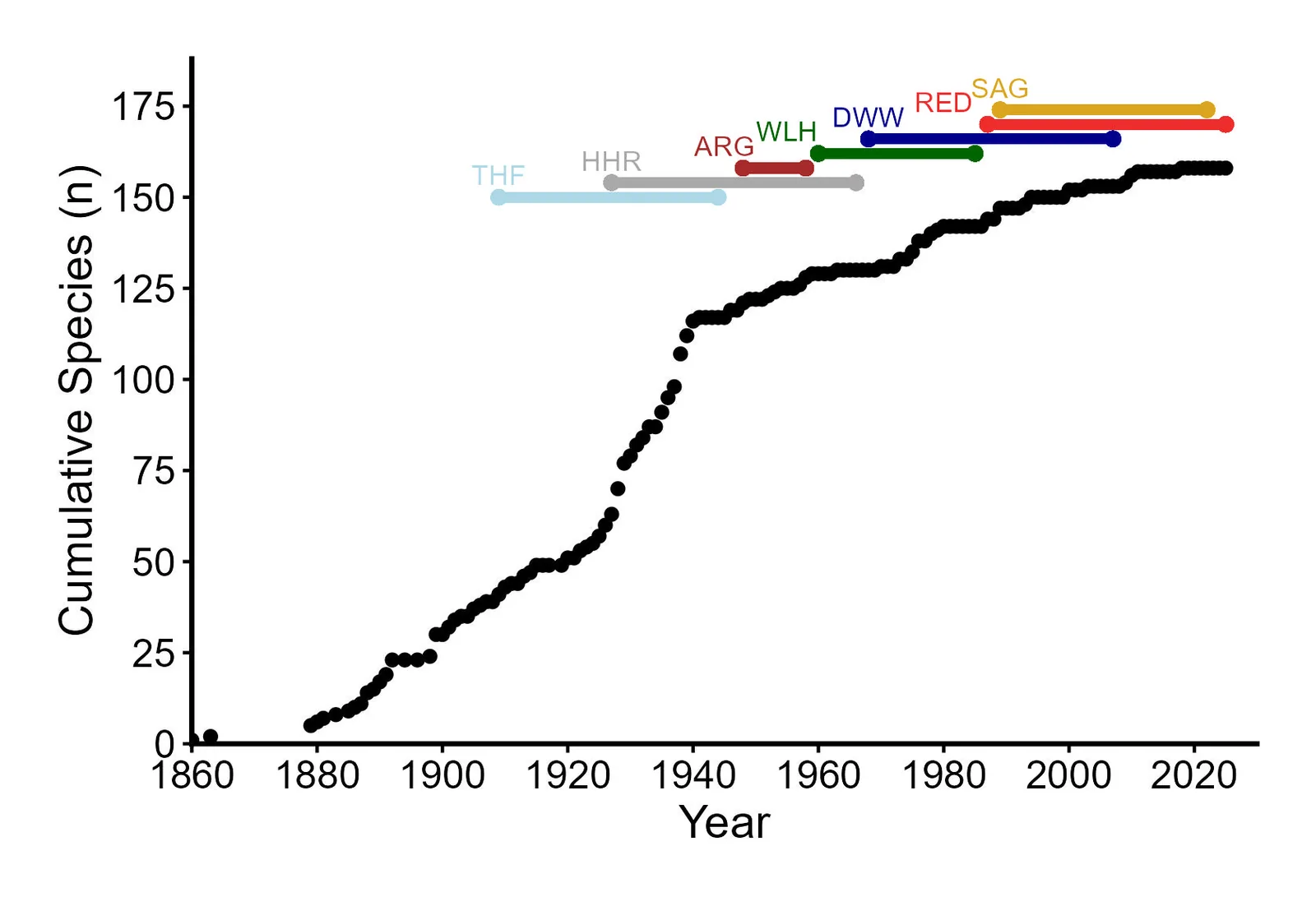
Cumulative species richness within the Midwest. Temporal span of collections made by several prolific collectors are indicated by horizontal bars and identified by the collector initials: Theodore H. Frison (THF), Herbert H. Ross (HHR), Arden R. Gaufin (ARG), William L. Hilsenhoff (WLH), Donald W. Webb (DWW), Ralph E. DeWalt (RED) and Scott A. Grubbs (SAG).

The vast majority of Walsh's insect collection was lost due to fire after his death (Tucker 1929). Several decades passed after Walsh (1862) before a young Theodore Frison of the Illinois Natural History Survey (INHS) initiated his work on Illinois stoneflies, first with the autumn and winter fauna (Frison 1929), prior to working on the state as a whole (Frison 1935, Frison 1937) (Fig. 1). Frison (1935) also noted that the INHS had been collecting in the Midwest states of Indiana, Michigan, Minnesota and Wisconsin. This broader Midwest perspective is obvious in Frison (1942), with several new species of Perlodidae described from Michigan. Frison died at age 50 in 1945 of a year-long illness (Gibson 1945), but his work on stoneflies had already sparked the interest of a new generation of plecopterologists. At roughly the same time, Philip Harden was a PhD student at the University of Minnesota. His first paper (Harden 1942) presented a faunal summary and taxonomic characteristics of the larval stages of several species known from Minnesota. His second and final treatment (Harden and Mickel 1952) has remained the only holistic work on Minnesota stoneflies to the present. Coincidental to Frison's prominent work in the 1940s, a young William Ricker took a teaching and research position at the University of Indiana in 1939 and remained there until 1950 (Stewart 2002). Ricker published the first checklist on Indiana stoneflies (Ricker 1945). Soon after, he followed with his important global treatment of Plecoptera (Ricker 1952) that included the descriptions of several new Midwest US species (e.g. *Amphinemura varshava* Ricker, 1952).

The first regional treatment of the Ohio fauna was accomplished by Walker (1947) for the unglaciated southeast of the state. Arden Gaufin (1956) expanded Walker's work westwards and northwards, but still covering only the southern half of the state. Gaufin's greatest contributions during his short stay in Ohio (through 1953, Fig. 1) was his collaborative, groundbreaking work on recognising that aquatic macroinvertebrates serve as excellent indicators of water quality conditions (Gaufin and Tazewell 1956).

The stonefly fauna of Wisconsin has been reasonably well known due to the research contributions of William Hilsenhoff during his long career at the University of Wisconsin. Like Gaufin, Hilsenhoff is best known for developing the first state-level macroinvertebrate-based biotic indices (Hilsenhoff 1977, Hilsenhoff 1982, Hilsenhoff 1987). Hilsenhoff's work on Wisconsin stoneflies resulted in several keys to genus and species, checklists of the state's fauna and autecological studies of Otter Creek of Sauk County and the Pine and Popple Rivers of north-eastern Wisconsin (Hilsenhoff 1970, Hilsenhoff and Narf 1972, Hilsenhoff and Billmyer 1973, Narf and Hilsenhoff 1974, Hilsenhoff 1975, Hilsenhoff 1995). He and his students also deposited nearly 6,000 vials of stoneflies, amounting to 27,702 Wisconsin specimens at the University of Wisconsin-Madison. Later, Stanley Szczytko of the University of Wisconsin-Stevens Point continued work on his magnum opus on eastern North American Isoperlinae (Szczytko and Kondratieff 2015) and mentored students on the life history and ecology of *Isoperla* Banks, 1906 occurring in Wisconsin (Jop and Szczytko 1984, Sandberg and Szczytko 1997). Though Szczytko never published a broad treatment of the Wisconsin stoneflies, he did amass 547 vials of material that have since been donated to the INHS and appear in this treatment.

During the 1970s and 1980s and aside from Hilsenhoff, there was relatively little other faunistic work on Midwest stoneflies, except for the revised state checklist of the Indiana fauna by Bednarik and McCafferty (1977), a checklist on north-eastern Minnesota Plecoptera (Lager et al. 1979) and several individual small watershed studies in Ohio (Tkac 1978, Robertson 1979, Tkac 1979, Robertson 1984, Beckett 1987, Fishbeck 1987). Much of the broader faunistic work on mid-western stoneflies was nested in taxonomic and systematic treatments of individual species, genera and families by Richard Baumann, Charlie Nelson, William Ricker, Herbert Ross, Bill Stark, Ken Stewart and Stan Szczytko. This was the commencement of a modern age with the eastern Nearctic fauna as a whole. Important revisionary systematic work continued through the 1980s and 1990s, together with individual and collaborative contributions also by Fred Kirchner, Boris Kondratieff, Riley Nelson and Rebecca Surdick.

The 1990s (and to the present) was a period of renewed interest in Illinois Plecoptera by INHS staff Don Webb, Mitchell Harris and R. Edward DeWalt. This was evidenced by state surveys for rare and vanishing populations (Webb and Harris 1993, Webb and DeWalt 1997, Webb 2002b) and, more importantly, starting to compare historic versus contemporaneous populations (Webb 2002a, DeWalt et al. 2005). Broader visions of addressing the Midwest fauna as a whole were taking shape at the time. Scott Grubbs from Western Kentucky University started working with Midwest stoneflies in 1994, mainly in Indiana and Michigan (e.g. Grubbs and Bright (2001), Grubbs (2004)) and began collaborating with DeWalt on several faunistic and systematic projects (DeWalt and Grubbs 2011, DeWalt et al. 2012, Grubbs and DeWalt 2012, Grubbs et al. 2012, Grubbs et al. 2013a, Grubbs et al. 2013b, Grubbs et al. 2014, DeWalt et al. 2016, Grubbs and DeWalt 2018). Mid-western specimens and data that had been accruing for several years were critical for optimising our treatments of individual species (e.g. *Perlesta ephelida* Grubbs and DeWalt 2012) and genera (e.g. *Prostoia* Ricker, 1952, Grubbs et al. (2014)). Iowa has been the mid-western state with the least degree of faunistic work on Plecoptera. Nonetheless, the recent treatments by Heimdal et al. (2004) and Heimdal and Birmingham (2006) have provided excellent additions to our understanding of the region as a whole. This is particularly apparent when studying regional east-west species in species richness.

Prior to this study, the seven states in the region were known to support the following stonefly species richness: Iowa = 44 (Heimdal et al. 2004, Heimdal and Birmingham 2006), Illinois = 79 (DeWalt et al. 2005, DeWalt and Grubbs 2011), Indiana = 93 (DeWalt and Grubbs 2011, Newman et al. 2021), Michigan = 68 (Grubbs et al. 2012), Minnesota = 54 (Harden and Mickel 1952, Lager et al. 1979), Ohio = 102 (DeWalt et al. 2012, DeWalt et al. 2016) and Wisconsin = 63 (Hilsenhoff and Narf 1972, Hilsenhoff and Billmyer 1973, Narf and Hilsenhoff 1974, Hilsenhoff 1995) for a total of 149 regional species (DeWalt et al. 2026).

### Dataset Description

**Museum Collections**. A large portion of the data used in this study resulted from examination of borrowed specimens from many institutional and private collections. Midwest institutions and others more distant maintain extensive collections of Midwest Plecoptera (Table 2). This is a direct result of the pioneering efforts of Theodore H. Frison (Frison 1929, Frison 1935), who set the standard for study in the region. Nearly all regional museum collections had been visited by Frison in the 1930s and 1940s, as evidenced by the substantial number of specimens preserved and determined by him. Frison's enthusiasm encouraged others to study stoneflies in their own states, often resulting in several hundred to well over a thousand vials, pins or lots being placed in collections. Most specimens within museums were not identified to the current taxonomic status of the field and required re-examination and identification. The specimen data source and number of records (# of vials or pins) are noted by the data provider (Table 2).

**Preparation of borrowed material**. Pinned adult stoneflies, some dating to as early as 1883, provided the oldest records and represented many geographic locations where species no longer occur. Pinned specimens accounted for 2.3% (n = 1,079) of the total occurrence records. Due to their shrivelled nature, these specimens required relaxing in a humid chamber for one to two days before examination. Capniidae, Leuctridae and Nemouridae could be identified after relaxing, based upon external genitalia. Others, such as males in the families Perlidae and Perlodidae, required additional work to evert the aedeagus and reveal sclerites and spinule patterns that are essential for species identification (Stark 2004, Szczytko and Kondratieff 2015). In these same families, females often have species-specific vaginal sclerites, partially sclerotized spermathecae and eggs with species-specific chorionic sculpturing that required preparation.

The abdomens of both sexes were clipped from the specimens at the second or third segment. Female terminalia with eggs were carefully rehydrated in distilled water until deformation was corrected, then transferred to 70% ethanol (EtOH). Association of eggs, abdomen and pins were maintained using a replicated number series such that identification numbers were affixed to pins and placed in watch glasses containing body parts. Using this approach, several specimens can be simultaneously prepared for examination. Abdomens were placed in 20 mm (inside diameter) Syracuse watch glasses containing cold 10% aqueous potassium hydroxide (KOH). Maceration of soft tissue proceeded for one or two hours for smaller species or as long as overnight for larger individuals.

Large male Perlidae (e.g. *Acroneuria* Pictet, 1841) are often identified based on aedeagal spinule patch patterns and shape and placement of lobes (Stark 2004). Most often, this structure is still within the abdomen in much the same arrangement as a rolled-up sock. In most cases, it is necessary to evert the aedeagus for confident identification. To extrude the aedeagus after KOH maceration, a curved probe was placed at the middle of the 10^th^ sternum to block tissue leaving via the anus. Using blunt forceps, pressure was simultaneously applied to the 9^th^ sternum forcing the central tube, distal sac and basal envelope of the aedeagus through the genital pore. The tube was then gently tugged through the surrounding envelope with forceps. The abdomen was replaced in KOH for up to 30 minutes for additional softening of the exposed bulbous middle of the aedeagus. This tube forms a narrow canal through which the sac of the aedeagus must past for full eversion. Careful squeezing and downward pressure on the distolateral end of the tube was used to create hydraulic pressure to push out the sac. Iterative soaking in KOH and squeezing may be necessary. Sometimes a blunt probe is use to stretch the opening of the tube. Once the aedeagus everts, the macerated tissue is flushed using a syringe and tap water. The action of the KOH is halted by transferring a few drops of household vinegar to the water directly above the abdomen. The vinegar was then rinsed from the specimen using a syringe and tap water. The abdomen was associated with the rest of the body by pinning a microvial with the tissue in a 50:50 mixture of glycerol and 70% EtOH.

Similar processes apply to small male perlids. For instance, the aedeagus of *Neoperla* Needham, 1905 consist of a fragile basal envelope, a variously sclerotised tube and basal bulb, and a long, thin sac or filament that often supports fields of cultriform spines on the surface (Stark 2004). The sac or filament is usually inside-out within the tube. Most of the necessary structures may be viewed through the integument after clearing, but in the *N. choctaw* group, the sac may need to be everted to reveal the pattern of cultriform spines. *Perlesta* Banks, 1906 males are much more difficult to study as pinned material. Positive identification usually requires live extrusion of the aedeagus (Grubbs and DeWalt 2018). Fortunately, the aedeagus of *Perlesta* may be seen through the cleared abdomen. Internally, the aedeagus resembles an inside-out sock. Leading from this bulbous aedeagus is a long filament (sac) that may be grabbed with a fine-tipped forceps and gently tugged to stretch the aedeagus to its full length. The structures needed for tentative identification are visible, albeit inside-out. If the regional species are known, then examination of paraprocts, the inside-out aedeagus and the female subgenital plate and eggs may yield a confident determination. Extruding the aedeagus of pinned *Isoperla* (Perlodidae) males is often challenging, but not impossible (Szczytko and Kondratieff 2015). Our methods follow that for large perlids as described above, often with a much reduced success rate. A series of specimens is extremely important for successful identification.

Fluid-preserved specimens accounted for over 93% (n = 44,126) of the total compiled records. Similar preparation efforts are used for wet specimens as for pinned. If specimen preservation is in 70—80% EtOH, then eversion of large perlid aedeagi is relatively simple. The same difficulties exist for fluid-preserved small perlids and *Isoperla* as for pinned specimens. Often, it was more efficient to clear alcohol specimens in hot KOH since cold maceration leaves them sticky. Specimens were heated in a double-boiler consisting of a 50 ml beaker (water) and medium shell vial (10% KOH) and were heated until boiling of the water in the beaker. Clearing may be conducted iteratively following initial observation. All specimens preserved in 95% EtOH are more brittle than those at lower concentrations and, hence, require more time and patience for proper extrusion.

**Collection of new specimens**. To augment the existing contemporary specimens present in museums, we embarked on a multi-season sampling programme that lasted from 2006 to 2025. Priority was given to historical locations for species and to the best remaining habitat in the region where complete historical assemblages of species may still exist. We also sampled many degraded streams to confirm suspected loss of species. Adults are often necessary for positive species-level identification. We used beating sheets, sweepnets, hand-picking from rocks, logs, bridges and buildings to collect adults during daylight hours. During warmer seasons, attended ultraviolet lights were placed on white, reflective sheets along streams and rivers. Adults that came to the lights were collected alive and aedeagi extruded under dissecting microscopes in hotel rooms or at the laboratory. It is inefficient to extrude the aedeagus using the naked eye and should be avoided. Most live specimens were briefly incapacitated in 50% EtOH immediately prior to eversion. Using fingers, gentle, slowly increasing pressure was applied to the abdomen until the basal envelope emerged from the 9^th^ sternum. Continued pressure usually resulted in emergence of the tube and sac (filament of *Perlesta* and some *Neoperla*). Often, it was necessary to use blunt forceps to gently squeeze the tube of *Neoperla* and *Perlesta* to fully evert the filament and caecum. Once the aedeagus was satisfactorily everted, forceps were used to hold pressure on the 9^th^ sternum and the specimen held in 50% EtOH for up to 1 min. Specimens were often then moved to 95% EtOH to ensure preservation of DNA. Other methods exist for fixing the extent of eversion in live specimens such as dipping the aedeagus in near boiling water for a few moments (Szczytko and Kondratieff 2015, Stark 2004).

Qualitative collections of nymphs using dipnets and hand-sorting from leaf packs, logs and rocks yielded many specimens. Habitats sampled included perched seeps, springs, springbrooks, headwater streams, the shorelines of large rivers and the wave-swept shores of the Laurentian Great Lakes. We estimate that at least 60% of the regional species cannot be identified to species using nymphs (Stewart and Stark 2002). This limitation includes the genera *Allocapnia* Claassen, 1928 and *Paracapnia* Hanson, 1946 (Capniidae), *Leuctra* Stephens, 1836 and *Zealeuctra* Ricker, 1952 (Leuctridae), *Amphinemura* Ris, 1902, *Ostrocerca* Ricker, 1952 and *Prostoia* (Nemouridae), some *Taeniopteryx* Pictet, 1841 (Taeniopterygidae), *Alloperla* Banks, 1906 and *Sweltsa* Ricker, 1943 (Chloroperlidae), some *Acroneuria*, all *Neoperla*, most *Perlesta* (Perlidae), some *Isoperla* (Perlodidae) and most specimens of early instars of *Pteronarcys* (Pteronarcyidae, but see Myers and Kondratieff (2017)). Pharate adults, those still contained within the nymphal skin, often may be identified as fully formed adults for those species with external genitalia.

Rearing of nymphs is another simple, reliable method to obtain adults for species-level identification (Stewart and Stark 2002). Live nymphs with darkened or enlarged wingpads were collected in the field and deposited into 12-ounce (0.35 litres) Styrofoam_TM_ cups with a small volume of stream water, mineral substrates and/or stream-conditioned leaf scraps as food for shredding species and a twig as an emergence site. A lid was affixed to the cup and a field notebook number, date and locality written on the top. Cups were stored in small insulated coolers, sometimes with a small amount of ice or snow. Winter emerging species could be kept in a cooler at near-freezing temperatures during fieldwork. Spring and summer species were kept at near stream temperatures from which they came, usually by placing ice in a plastic bag inside a cooler. In the laboratory, specimens were placed into a Frigid Units Living Stream_TM_ (a large tank and pump with a condenser) with cups modified with mesh and the field lids transferred. Cups were suspended on Styrofoam_TM_ sheets in the flow of water. Water temperature mimicked source stream temperature. Adults of perlids and perlodids were often fed chironomid midges, blackflies and small hydropsychid caddisflies. Adult specimens were removed daily with their exuviae and kept in small, ventilated bottles until they darkened. The aedeagus was then everted as already described. Adults of darkened Capniidae, Leuctridae, Nemouridae, Peltoperlidae, Pteronarcyidae and Taeniopterygidae could be immediately preserved with the exuviae.

While several of us have preserved newly-collected specimens in 95% EtOH, these specimens are often brittle. We have now adopted the use of 70—80% EtOH to preserve specimens for one or two days. This allows examination of supple specimens that are fully extended. These specimens may be further dehydrated in 95% EtOH after a few days and their use for molecular studies remains well protected.

**Agency data**. We also accepted specimen data from two state agencies (Ohio Environmental Protection Agency and Hygienics Laboratory of Iowa) that conduct biological monitoring using aquatic macroinvertebrates. The inclusion of agency data was warranted because they sampled in spring and summer and often included species-level identifications as the result of hiring highly trained taxonomists.

### Data Methods

Occurrence data were compiled into a Filemaker Pro or a TaxonWorks (https://docs.taxonworks.org) dataset. Data were standardised to a Darwin Core Archive occurrence dataset extension (https://dwc.tdwg.org/list/#dwc_Occurrence). We associated most specimens with their database record using a paper catalogue number--a unique identifier as described in DeWalt et al. (2018). Unfortunately, this was not the case for OEPA specimens, Iowa Hygienics Laboratory specimens, Western Kentucky University material and literature sources. As is often the case for many small collections and institutions, multiple agencies have yet to adopt the use of catalogue numbers. Specimen data were gathered in accordance with DeWalt et al. (2018) wet collection protocols. Names used as determinations (as OTUs) were kept distinct from valid names provided from Plecoptera Species File (DeWalt et al. 2026).

Most location labels printed prior to the year 2000 did not contain geographic coordinates. We georeferenced these locations using Acme Mapper (2026) (datum WGS-84). This programme provides 1:24000 scale topographic maps, satellite and road map coverages of the USA that ensure the greatest possibility of finding complex locations. Collector-provided coordinates were projected to verify that they accurately matched label descriptions. Where they did not match, coordinates were corrected. We used a decimal degree format, most often to five significant figures, to improve the usability of the data by others. Estimated precision is presented as a radius from the coordinate pair location in metres. A map that contains all unique collection locations overlaid on United States Geological Survey Hierarchical Unit Code eight (USGS HUC-8, 316 drainages; United States Geological Survey (2019)) scale drainages was constructed using ArcGIS Pro (Version 3.6) and projected to Universal Transverse Mercator Zone 16 North (EPSG: 32617) for visualisation.

Specimen occurrence records were cleaned, organised and standardised using Microsoft Excel (Version 2601), RStudio (Version 4.3.0) and OpenRefine (Version 3.7.2). All specimen data have been milled through Pensoft's Integrated Publishing Tool to produce a Darwin Core Archive (DwC-A) file that allows indexing of the data with the Global Biodiversity Information Facility (GBIF) and TaxonWorks.

All georeferenced specimen records with coordinate uncertainty of less than 10,000 m were associated with USGS Hydrologic Unit Code 8 (HUC-8) drainage basins. A binary presence-absence data matrix of species by HUC-8 drainage was constructed for the entire Midwest region. To determine adequacy of sampling within the entire region, a species accumulation curve using three non-parametric species richness estimators (Chao2, Bootstrap and Jackknife 1) was calculated from the presence-absence matrix using the R package *vegan* (Oksanen et al. 2026), then plotted against HUC-8s with the cumulative assemblage richness (Sobs-Mao Tau).

## Geographic coverage

### Description

**Figure 2.**
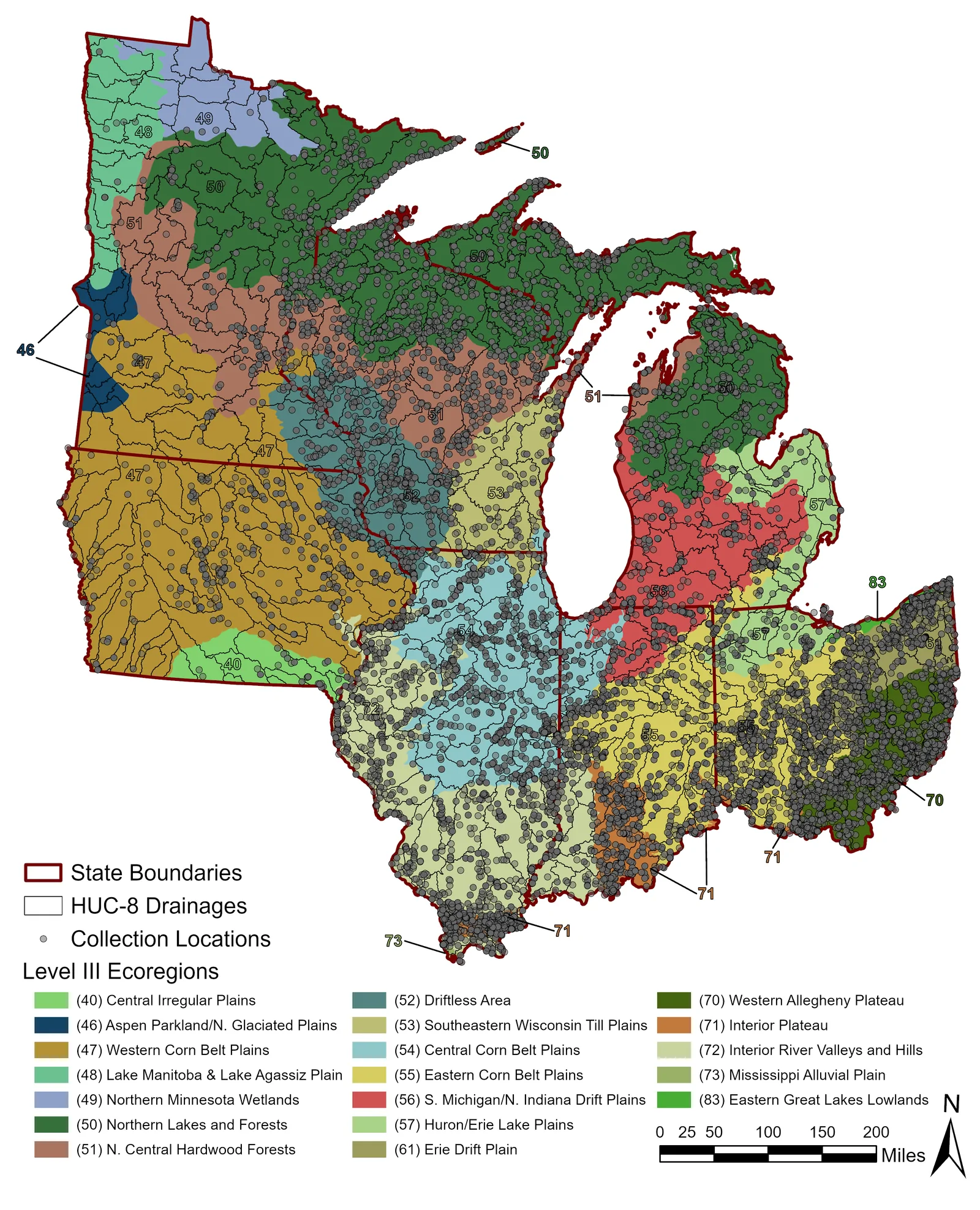
All collection locations of stoneflies in the Midwest overlaid on US state boundaries (red), US EPA Level III Ecoregions and USGS HUC-8 drainage basin outlines (black).

Within the study region, collections have occurred in 91.4% of counties (570/623) and 91.1% of HUC-8 (288/316) drainage basins. Illinois has the greatest number of occurrence records (n = 11,072), followed by Ohio (n = 9,667), Wisconsin (n = 9,520), Indiana (n = 7,947), Michigan (n = 5,412), Minnesota (n = 3,496) and Iowa (n = 1,932). Despite near-complete sampling of HUC-8 drainages and counties, the density of occurrence records within the study region is unevenly distributed. Occurrence records are greatest in the southern and eastern parts of the study region, with diminishing sampling completeness in western areas of Iowa and Minnesota (Fig. 2). All 28 unsampled HUC-8 drainage basins and counties are found within western Iowa and Minnesota. The geographic bias within this dataset results from the discrepancy between states with a long history of collecting by taxonomic experts stationed at local institutions, the relative remoteness and inaccessibility of the western reaches of the region and collecting bias towards public lands and uplifted regions.

**Figure 3.**
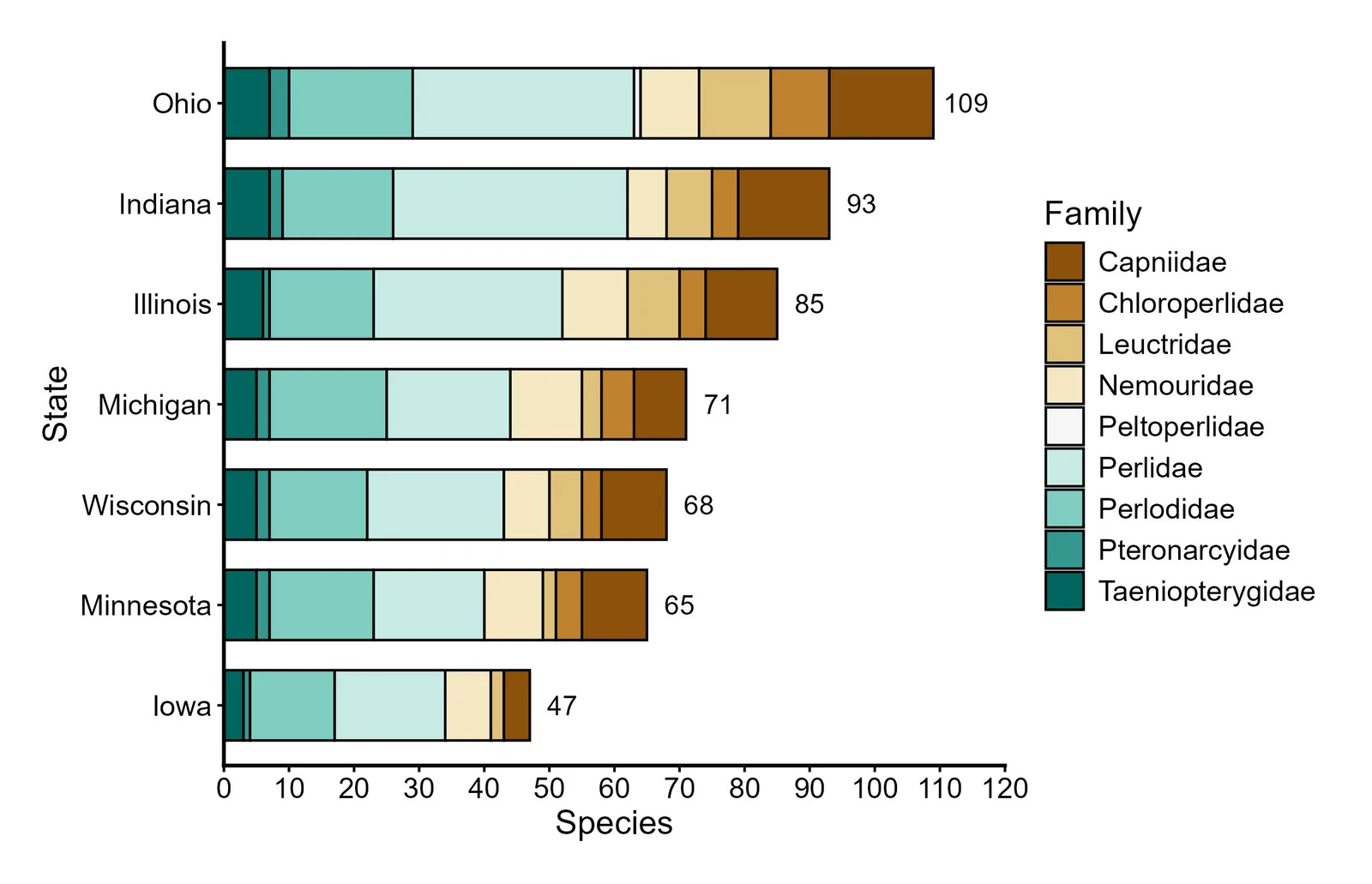
Species richness per state colour-coded by family.

Family richness between states varied little, with each state's fauna composed of eight families, with the exception of Iowa and Ohio. Iowa had the lowest family richness with only seven families present, whereas Ohio had the greatest family richness of the Midwest region with all nine eastern Nearctic families present. The family Peltoperlidae was only recovered in Ohio. The additional family not recovered from Iowa was Chloroperlidae. Species richness within each state follows an east-west and north-south gradient with the greatest number of species now reported from Ohio (n = 108), followed by Indiana (n = 92), Illinois (n = 85), Michigan (n = 71), Wisconsin (n = 68), Minnesota (n = 66) and Iowa (n = 47) (Fig. 3). Of the 157 species known from the region, 37 were found in only a single state (Table 3). Ohio had the greatest number of unique species present, with 24 species not shared with the remaining Midwest states. This is followed by Illinois (n = 6), Minnesota (n = 4), Michigan (n = 2) and Indiana (n = 1). Neither Wisconsin nor Iowa had any unique species. In addition, 28 and 33 species were found in two and three states, respectively. Another 23 species were ubiquitous throughout the region and were found in all seven states.

### Coordinates

36.98787 and 48.77548 Latitude; -96.9374 and -80.5283 Longitude.

## Taxonomic coverage

### Description

**Figure 4.**
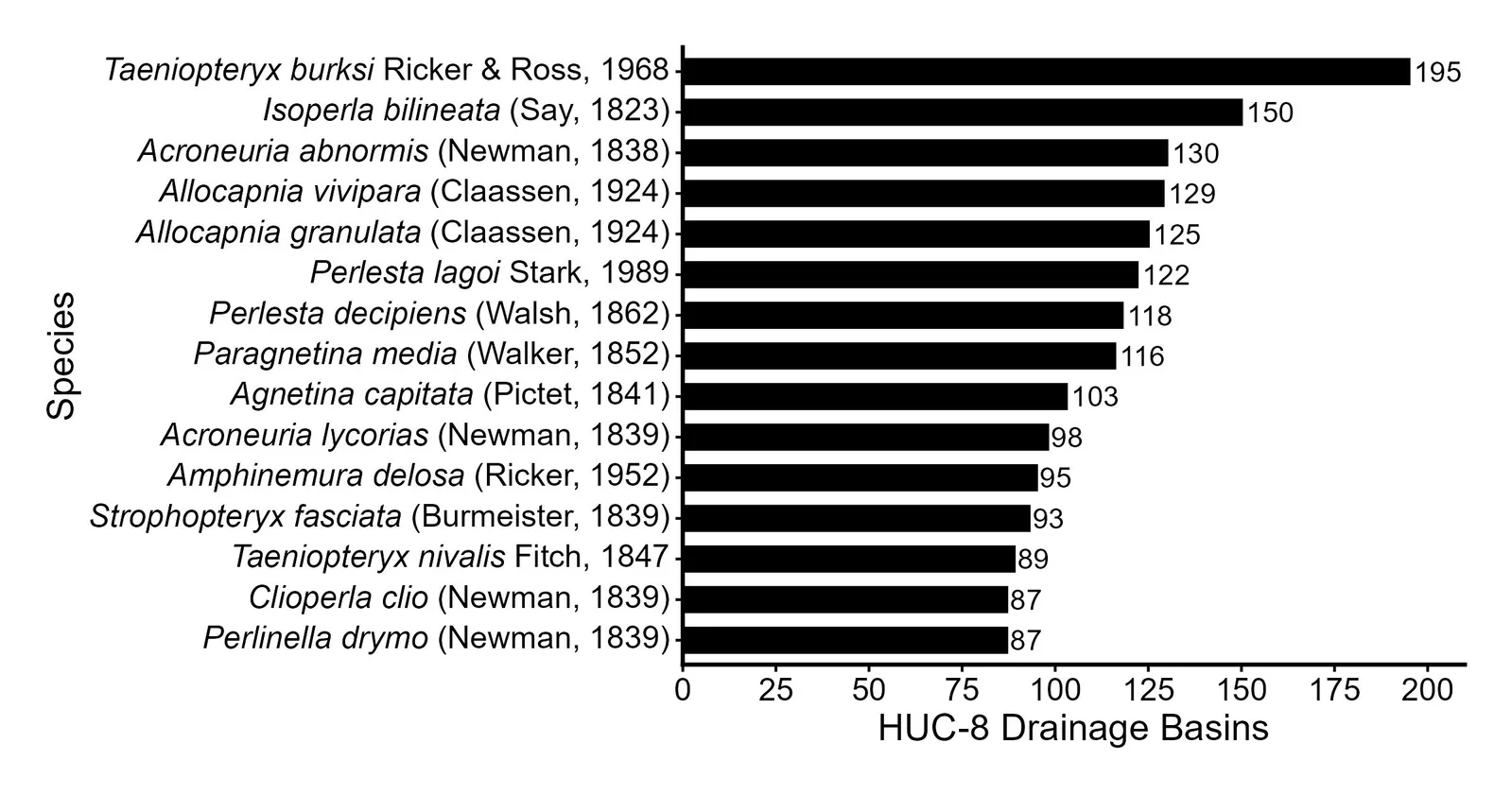
The 15 most prevalent stonefly species in the Midwest USA by decreasing order of unique USGS Hydrologic Unit Code 8 (HUC-8) drainage basins occupied.

This dataset encompasses 157 species from 41 genera and nine families (Table 3). Of the total species, 156 species are currently accepted (DeWalt et al. 2026). An additional species is awaiting future description. Within this dataset, 44,508 records are determined to species, 4,440 to genus, 24 to family and 22 to order. An additional 52 records included subspecies designations. The greatest number of occurrence records per family was from Perlidae (n = 15,298). This was followed by Perlodidae (n = 10,108), Capniidae (n = 9,946), Taeniopterygidae (n = 5,036), Nemouridae (n = 3,563), Leuctridae (n = 1,949), Pteronarcyidae (n = 1,562), Chloroperlidae (n = 1,522) and Peltoperlidae (n = 40). The greatest number of occurrence records by genus was for *Allocapnia* (Capniidae, n = 9,402), *Isoperla* (Perlodidae, n = 8,122), *Acroneuria* (Perlidae, n = 5,407), *Perlesta* (Perlidae, n = 5,000) and *Taeniopteryx* (Taeniopterygidae, n = 4,168). The 15 most prevalent species in this dataset comprise 51% of the total species-level occurrence records. The five most prevalent species were *Allocapnia vivipara* (Claassen, 1924) (Capniidae, n = 3,257), *Taeniopteryx burksi* Ricker & Ross, 1968 (Taeniopterygidae, n = 2,533), *Acroneuria frisoni* Stark & Brown, 1991 (Perlidae, n = 1,967), *Allocapnia granulata* (Claassen, 1924) (Capniidae, n = 1,965) and *Isoperla bilineata* (Perlodidae, n = 1,603). With the exception of *A. frisoni*, the species with the greatest number of occurrence records were also found across the most widespread geographic ranges within the Midwest (Fig. 4). Of these species, *T. burksi* was found in 61% of all HUC-8 catchments. Alternatively, 82 species are known from fewer than 100 occurrence records. Of these, 30 species are known from < 25 records and an additional nine species were known from a single record.

**Figure 5.**
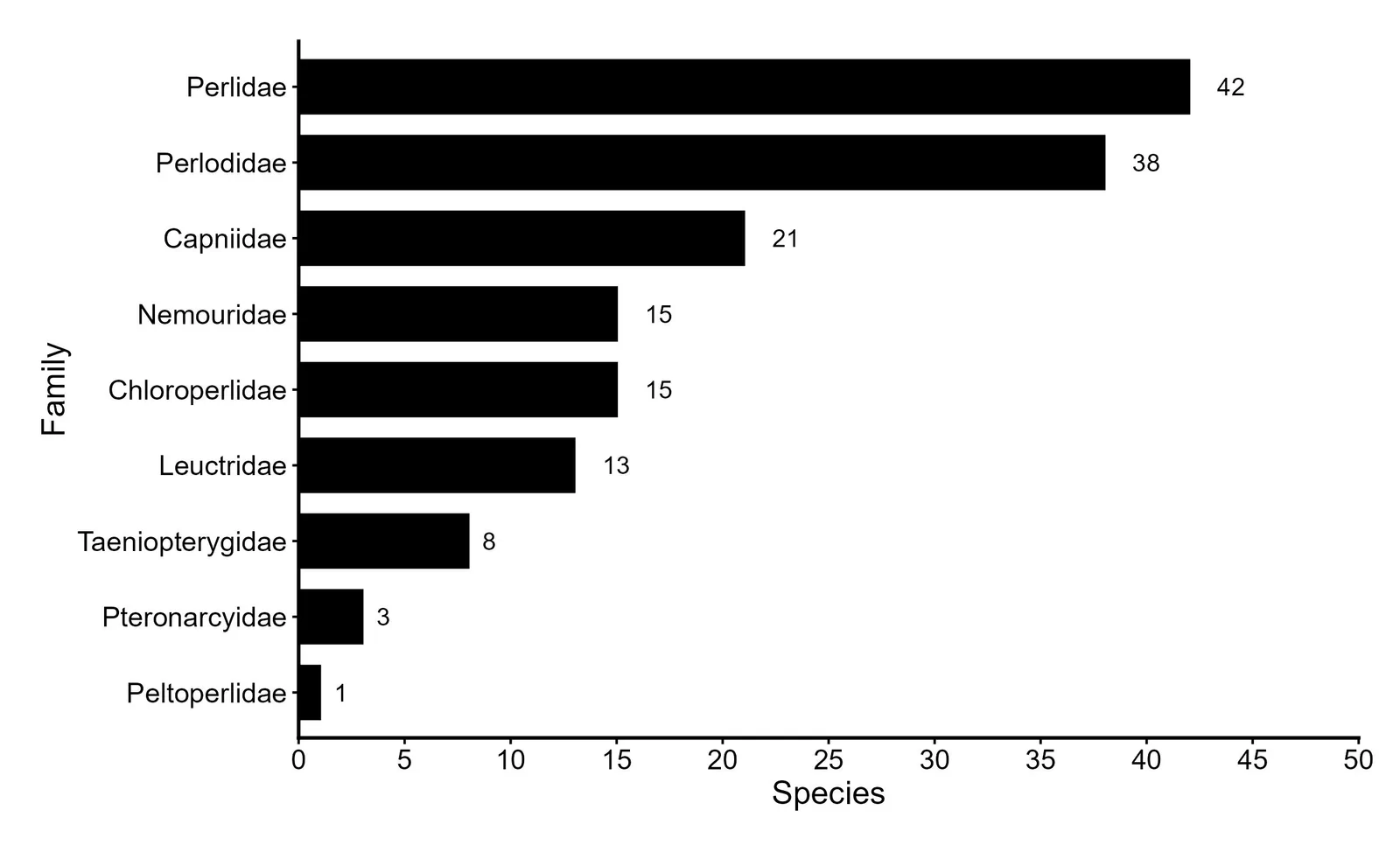
Species richness by family of stoneflies in the Midwest, USA.

The Midwest stonefly fauna represents 20% of the total described, extant Nearctic species richness (DeWalt et al. 2026). The most species-rich family, Perlidae, comprises 42 species, followed by Perlodidae (38 species), Capniidae (21 species), Chloroperlidae (15 species) and Nemouridae (15 species) (Fig. 5). These five families contain 84% of the regional fauna. The remaining four families, Leuctridae (13 species), Taeniopterygidae (8 species), Pteronarcyidae (3 species) and Peltoperlidae (1 species), host the remaining 25%. The most diverse genera within the Midwest are *Isoperla* (Perlodidae, 24 species), *Allocapnia* (Capniidae, 16 species), *Perlesta* (Perlidae, 12 species), *Acroneuria* (Perlidae, 11 species), *Alloperla* (Chloroperlidae, 11 species) and *Neoperla* (Perlidae, 10 species). Of the regional fauna, 18 genera were represented by a single species. Occurrence records for an undescribed species with a temporary name assigned, *Perlesta* WI-1, are included in this dataset as this species is supported by both morphological and molecular evidence (South et al. 2019).

**Figure 6.**
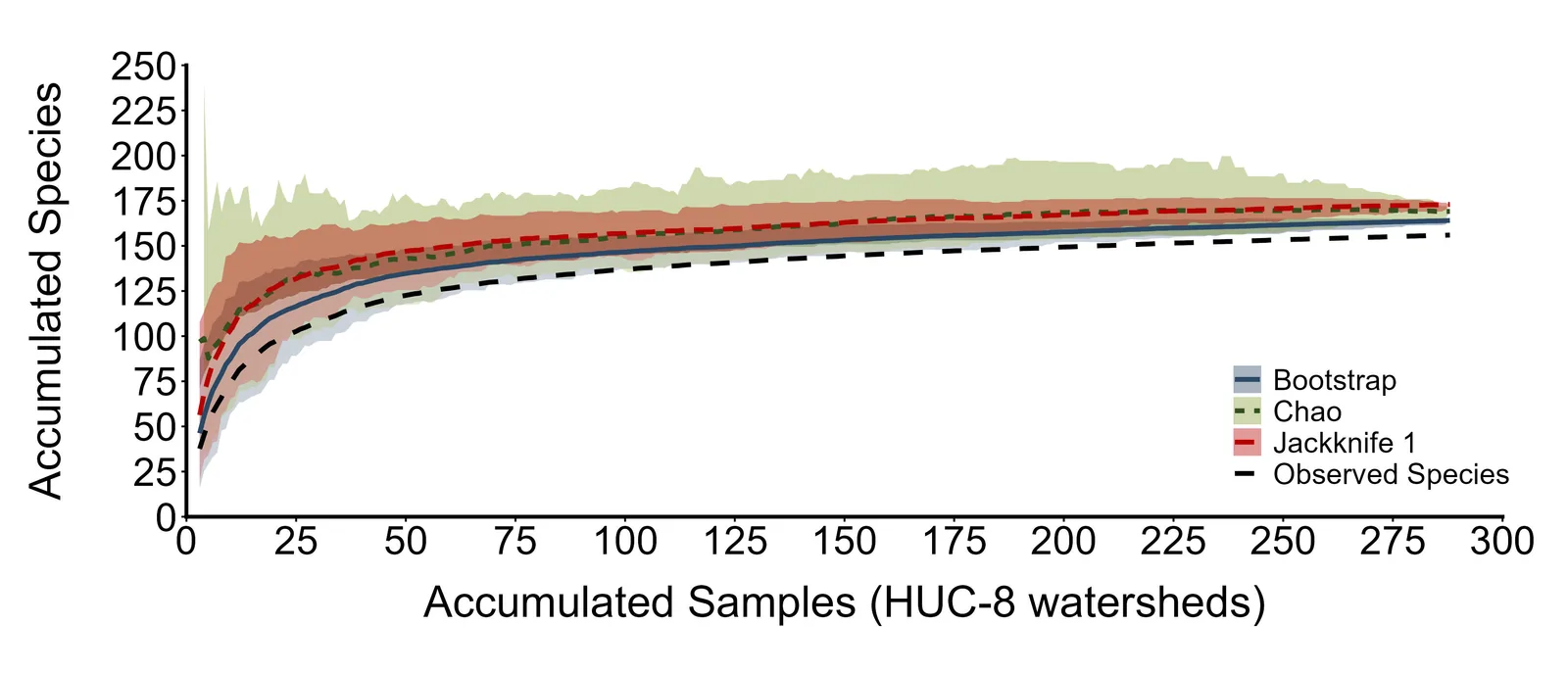
Estimates of Midwest, USA stonefly species richness from Bootstrap, Chao2 and Jackknife 1 estimators across USGS Hydrologic Unit Code 8 (HUC-8) drainage basins with the number of observed species indicated.

Estimates of species richness at the regional level indicate that additional species are predicted to occur in the study region despite nearly two centuries of intensive collecting efforts. Estimates of total species richness for the Midwest range from 164 (± 2.5) to 172 (± 4.9) from the bootstrap and first-order jackknife estimators, respectively (Fig. 6). These species richness estimates for the Midwest indicate an additional eight to 16 species may be recovered from additional collecting effort in undersampled subregions or reassessment of specimens from genera that have recently undergone taxonomic revision. As the Bootstrap estimator is a conservative, non-parametric method and not strongly influenced by rare species within community data (Walther and Morand 1998), the richness value provided (164 ± 2.5) may be the most accurate for the seven-state region. The recent contributions of additional state records to the known fauna of well-sampled states, such as Illinois (*Ostrocerca truncata* (Claassen, 1923), Grubbs and Baumann 2023) and Ohio (*Isoperla kirchneri* Szczytko & Kondratieff, 2015, examined BYUC material), illustrate how additions to the regional fauna can occur despite a long history of collecting and further demonstrate the importance of refining distributional knowledge through targeted sampling.

## Temporal coverage

**Data range:** 1860-1-01 – 2025-6-14.

### Notes

Although the first reports of Midwest stoneflies originate from the early 19^th^ century (Say 1823), the earliest archived physical specimens available for study were from 1860 (Fig. 1). Early records of stoneflies within the region are sparse, with only 0.3% (n = 144) of all records collected prior to 1900. The number of species known from the region rapidly increased during several periods, most notably during the collections of the 1940s through the 1960s (Fig. 1). Despite the numerous records collected during the early and middle 20^th^ century, over half (51%, n = 23,683) of the occurrence records were collected after 1990. This increase reflects increased attention to the Midwest stonefly fauna in an attempt to document faunal shifts (Webb 2002a, DeWalt et al. 2005, Grubbs et al. 2013a).

The long-term nature of this dataset, in conjunction with recent targeted collections of uncommon species throughout the study region, increases our confidence in identifying species as extirpated regionally. The presence of 13 species in the Midwest, 8% of the regional fauna, is based solely upon historical material collected before 1980. Ten species have not been recollected since 1970, nine of which have not been recorded since before 1960 (Table 4). Four of these species (*A. roberti* Surdick, 1981, *I. conpsicua* Frison, 1935, *I. emarginata* Harden & Mickel, 1952 and *I. maxana* Harden & Mickel, 1952) are presumed extinct. One species, *Neoperla mainensis* Banks, 1948, has not been recollected from across its broader distribution since 1944 and has likely undergone severe range reductions over the past century (Stark and Baumann 1978, DeWalt, unpublished data). The remaining species are known from broader distributions but, with the exception of *I. kirchneri*, are likely fully extirpated from the Midwest. The latter species was only described recently and may be more common, especially in Ohio, than presently understood. The larva of this species is currently undescribed, making positive determinations of this species problematic. The lack descriptions of the many new Isoperla species, described by Szczytko & Kondratieff (2015), is slowly being remedied with careful integrative analyses (e.g. Grubbs et al. (2025)).

The absence of contemporary records for multiple species suggests that all Midwest states have lost stonefly fauna to varying degrees of severity. Within individual states, Illinois has experienced the greatest loss of stoneflies, with 18 (21%) species last recorded prior to 1980, of which 16 (19%) species were last recorded prior to 1970 (Table 3). Following Illinois, the number of species not recovered within individual states after 1970 was next greatest for Indiana (n = 9, 10%), Michigan (n = 8, 11%), Ohio (n = 8, 7%), Minnesota (n = 7, 11%), Wisconsin (n = 2, 3%) and Iowa (n = 1, 2%). Using 1980, a less conservative threshold to define presumed extirpated species, the greatest losses following Illinois were found in Minnesota (n = 13, 20%), Ohio (n = 12, 11%), Indiana (n = 10, 11%), Michigan (n = 8, 12%), Wisconsin (n = 4, 6%) and Iowa (n = 2, 4%). Using the number of state extirpations as a proxy of range contractions, the greatest number of species lost by family using the conservative threshold of the year 1970 was found in the family Perlidae (n = 17, 40%), followed by Perlodidae (n = 8, 21%), Capniidae (n = 5, 24%), Chloroperlidae (n = 3, 20%), Leuctridae (n = 2, 15%), Pteronarcyidae (n = 1, 33%) and Nemouridae (n = 1, 7%). By family, the number of species only collected prior to 1980 followed a similar trend and was greatest in Perlidae (n = 20), Perlodidae (n = 10), Capniidae (n = 7), Chloroperlidae (n = 5), Leuctridae (n = 4), Nemouridae (n = 3) and Pteronarcyidae (n = 1). All species of Peltoperlidae and Taeniopterygidae were recovered during contemporaneous collections.

Overall, stonefly diversity over the course of nearly two centuries within the Midwest has been reduced across states and families, as is evident by the multiple species absent from the contemporary fauna despite recorded historical presence and intensive collection efforts across the region. Determining shifting diversity patterns was only enabled by the review of historical data in conjunction with contemporary collecting efforts. We provide the first comprehensive summary of the Midwest stonefly fauna to date, based upon the compilation, review and databasing of specimen data from multiple sources, including natural history museums, state agencies, reliable literature and contemporary collecting efforts. The utility of compiling both historical and contemporary collection data in addressing species' temporal and spatial distribution dynamics is demonstrated, but will be addressed in greater detail through future manuscripts utilising these data. These occurrence data compiled from archived specimens in natural history museums were integral in identifying the historical presence of species from which modern comparisons could be derived. The importance of natural history museums and the archived specimens held within cannot be overemphasised in the importance of creating this dataset. Occurrence datasets with long temporal coverage and broad geographic scale, based upon the review of specimen data archived in natural history museums, allow for accurate assessment of regional endemism and diversity hotspots. As part of a broader trend of refining current distributional knowledge of North American stoneflies through updated taxonomy and faunistic datasets, this Midwest treatment dataset is the first step in this long-term goal towards a continental understanding of stonefly richness and diversity patterns. Following a similar methodological approach, a parallel multi-state regional treatment is currently under preparation for the Northeast, USA. Like the Midwest, the Northeast region shares both a rich history of stonefly taxonomy coupled with a modern, renewed interest in thorough contemporary sampling efforts. Identification of species' historical presence will help guide conservation decisions for effective prioritisation and management. Landscape-scale treatments of regional fauna are integral components in understanding the dynamics of richness and diversity patterns, both spatially and temporally, through techniques such as distribution and occupancy modelling.

## Usage licence

### Usage licence

Creative Commons Public Domain Waiver (CC-Zero)

## Data resources

### Data package title

Stoneflies (Insecta: Plecoptera) of the Midwest, USA: A Species Occurrence Dataset Compiled over Nearly 170 Years

### Resource link

https://doi.org/10.15468/d347ck

### Number of data sets

1

### Data set 1.

#### Data set name

Midwest USA Plecoptera

#### Data format

Darwin Core Archive Format

#### Download URL

https://ipt.pensoft.net/archive.do?r=midwestern_usa_plecoptera

#### Description

The following dataset, "Stoneflies (Insecta: Plecoptera) of the Midwest, USA: A Species Occurrence Dataset Compiled over Nearly 170 Years", is a species occurrence dataset of reviewed and databased stonefly specimens collected from within the Midwest, USA that are vouchered in natural history museums and state water quality monitoring agencies. Additional records have been incorporated from trusted literature sources and contemporary collecting efforts. This dataset comprises 49,046 occurrence records representing 221,334 specimens and 156 valid species and one additional undescribed species is included. These occurrence data are formatted in a comma-separated matrix (.csv) file and have been normalised to meet Darwin Core Archive standards. We use the word "record" here to mean a single occurrence of a specific lifestage and sex of a determined taxonomic unit from a single collecting event. This means a single species could have multiple records for the same collecting event if multiple sexes or life stages were collected.

These data have also been made available within this manuscript as a compressed, supplementary file to provide additional access options (Suppl. material 1). We ask that any usage of this dataset include the appropriate attribution in the form of the dataset citation generated by GBIF.

**Data set 1.** 

Column label	Column description
accessRights	Information about who can access the resource or an indication of its security status. http://purl.org/dc/terms/accessRights
basisOfRecord	The specific nature of the data record. https://rs.tdwg.org/dwc/terms/basisOfRecord
associatedReferences	A list (concatenated and separated) of identifiers (publication, bibliographic reference, global unique identifier, URI) of literature associated with the dwc:Occurrence. http://rs.tdwg.org/dwc/terms/associatedReferences
catalogNumber	An unique identifier for the record within the dataset or collection. https://rs.tdwg.org/dwc/terms/catalogNumber
catalogNumberPrefix	Internal dataset value specifying the institution code applying the catalogue number, when applicable.
occurrenceRemarks	Comments or notes about the dwc:Occurrence. http://rs.tdwg.org/dwc/terms/occurrenceRemarks
class	The full scientific name of the class in which the dwc:Taxon is classified. https://rs.tdwg.org/dwc/terms/class
continent	The name of the continent in which the collecting event location occurs. https://rs.tdwg.org/dwc/terms/continent
coordinateUncertaintyInMetres	The horizontal distance (in metres) from the given decimalLatitude and decimalLongitude describing the smallest circle containing the whole of the collecting event location. The value is left empty if the uncertainty is unknown, cannot be estimated or is not applicable (because there are no coordinates). https://rs.tdwg.org/dwc/terms/coordinateUncertaintyInMeters
country	The name of the country or major administrative unit in which the collecting event location occurs. https://rs.tdwg.org/dwc/terms/country
countryCode	The standard code (ISO 3166-1-alpha-2) for the country in which the collecting event location occurs. https://rs.tdwg.org/dwc/terms/countryCode
county	The full, unabbreviated name of the next smaller administrative region than stateProvince (county, shire, department etc.) in which the collecting event location occurs. https://rs.tdwg.org/dwc/terms/county
dataSetName	The name identifying the data set from which the record was derived. http://rs.tdwg.org/dwc/terms/datasetName
dateIdentified	The date on which the subject was determined as representing the dwc:Taxon. https://rs.tdwg.org/dwc/terms/dateIdentified
day	The integer day of the month on which the collecting event occurred. https://rs.tdwg.org/dwc/terms/day
decimalLatitude	The geographic latitude (in decimal degrees, using the spatial reference system given in dwc:geodeticDatum) of the geographic centre of a collecting event location. http://rs.tdwg.org/dwc/terms/decimalLatitude
decimalLongitude	The geographic longitude (in decimal degrees, using the spatial reference system given in dwc:geodeticDatum) of the geographic centre of the collecting event location. https://rs.tdwg.org/dwc/terms/decimalLongitude
endDayOfYear	The latest integer day of the year on which the collecting event occurred. http://rs.tdwg.org/dwc/terms/endDayOfYear
eventDate	The date-time or interval during which a collecting event occurred in ISO 8601 format. https://rs.tdwg.org/terms/eventDate
eventDateEnd	Internal dataset value of indicating the date when the collecting event ended in ISO 8601 format.
family	The full scientific name of the family in which the taxon is classified. https://rs.tdwg.org/terms/family
fieldNumber	An identifier given to the collecting event in the field serving as a link between field notes and the collecting event. https://rs.tdwg.org/dwc/terms/fieldNumber
genus	The full scientific name of the genus in which the taxon is classified. https://rs.tdwg.org/dwc/terms/genus
geodeticDatum	The ellipsoid, geodetic datum or spatial reference system (SRS), upon which the geographic coordinates given in decimalLatitude and decimalLongitude are based. https://rs.tdwg.org/dwc/terms/geodeticDatum
georeferencedBy	A person, group or organisation who determined the georeference (spatial representation) for the collecting event location. https://rs.tdwg.org/dwc/terms/georeferencedBy
georeferenceRemarks	Comments or notes about the spatial description determination, explaining assumptions made in addition or opposition to the those formalised in the method referred to in georeferenceSource. https://rs.tdwg.org/dwc/terms/georeferenceRemarks
georeferenceSources	A map, gazetteer or other resource used to georeference the collecting event location. https://rs.tdwg.org/dwc/terms/georeferenceSource
habitat	A category or description of the habitat in which the collecting event occurred. https://rs.tdwg.org/dwc/terms/habitat
higherClassification	A list of taxa names terminating at the rank immediately superior to the referenced taxa. http://rs.tdwg.org/dwc/terms/higherClassification
identificationQualifier	A controlled value to express the determiner's doubts about the taxa identification. https://rs.tdwg.org/dwc/identificationQualifier
identifiedBy	A person, group or organisation who assigned the taxa name to the subject. http://rs.tdwg.org/dwc/terms/identifiedBy
identifiedbyID	A list (concatenated and separated) of the globally unique identifier for the person, people, groups or organisations responsible for assigning the taxon name to the subject. http://rs.tdwg.org/dwc/terms/identifiedByID
individualCount	The number of individuals present at the time occurrence. https://rs.tdwg.org/terms/individualCount
infraspecificEpithet	The name of the lowest or terminal infraspecific epithet of the scientificName, excluding any rank designation. http://rs.tdwg.org/dwc/terms/infraspecificEpithet
institutionCode	The name or acronym in use by the institution having custody of the object(s) or information referred to in the record. http://rs.tdwg.org/dwc/terms/institutionCode
institutionID	An identifier for the institution having custody of the object(s) or information referred to in the record. http:rs.tdwg.org/dwc/terms/institutionID
kingdom	The full scientific name of the kingdom in which the taxa is classified. http://rs.tdwg.org/dwc/terms/kingdom
verbatimLocality	The original textual description of the place. http://rs.tdwg.org/dwc/terms/verbatimLocality
lifeStage	The age class or lifestage of the specimens at the time the occurrence was recorded. https://rs.tdwg.org/dwc/terms/lifeStage
locality	The specific description of the place the collecting event occurred. https://rs.tdwg.org/dwc/terms/locality
maximumElevationInMeters	The upper limit of the range of elevation above sea level, in metres. http://rs.tdwg.org/dwc/terms/maximumElevationInMeters
vernacularName	A common or vernacular name. http://rs.tdwg.org/dwc/terms/vernacularName
waterBody	The name of the waterbody in which the dcterms:Location occurs. http://rs.tdwg.org/dwc/terms/waterBody
MID_Decade	Internal dataset value corresponding to ten year increments beginning in 1860 in which the collecting event date occurs.
MID_HUC8	The 8 digit code corresponding to the USGS Hydrologic Unit Code 8 watershed in which the collecting event location occurs.
MID_HUC8_NAME	The vernacular name corresponding to the USGS Hydrologic Unit Code 8 watershed in which the collecting event location occurs.
MID_Lvl_I_Eco_KEY	Internal dataset value specifying which US EPA Level I Ecoregions the collecting event occurs in.
MID_Lvl_II_Eco_KEY	Internal dataset value specifying which US EPA Level II Ecoregions the collecting event occurs in.
MID_Lvl_III_Eco_KEY	Internal dataset value specifying which US EPA Level III Ecoregions the collecting event occurs in.
MID_Period_1960	Internal dataset value indicating if the collecting event occurred prior to the year 1960.
MID_Period_1970	Internal dataset value indicating if the collecting event occurred prior to the year 1970.
MID_precisionCode	Internal dataset value, codified to be analogous to coordinateUncertaintyInMeters.
MID_publicLandName	The name of the publicly accessible land held in trust by organisations or federal, state or local governments in which the collecting event location occurs.
MID_recordID	Internal dataset value for organisation and sorting.
MID_season	The season (i.e. winter, spring, summer or autumn) in which the collecting event date occurs. Determined using the endDayOfYear field.
MID_streamWidth	Internal dataset value codified to represent each of the stream width categories in streamWidthRangeInMeters.
MID_streamWidthRangeInMeters	Internal dataset value for an estimated width between banks of the waterbody in which the collecting event location occurs.
minimumElevationInMeters	The lower limit of the range of elevation (above sea level) in metres. https://rs.tdwg.org/dwc/terms/minimumElevationInMeters
month	The integer month in which the collecting event occurred. https://rs.tdwg.org/dwc/terms/month
occurrenceID	A globally unique identifier for the occurrence record. http://rs.tdwg.org/dwc/terms/occurrenceID
occurrenceStatus	A statement about the presence or absence of a taxon at a location. http://rs.tdwg.org/dwc/terms/occurrenceStatus
order	The full scientific name of the order in which the taxon is classified. http://rs.tdwg.org/dwc/terms/order
otherCatalogNumbers	A list (concatenated and separated) of previous or alternative fully qualified catalogue numbers or other human-used identifiers for the same occurrence record, whether in the current or any other data set or collection. http://rs.tdwg.org/dwc/terms/otherCatalogNumbers
year	The four-digit year in which the dwc:Event occurred, according to the Common Era Calendar. http://rs.tdwg.org/dwc/terms/year
phylum	The full scientific name of the phylum in which the taxon is classified. http://rs.tdwg.org/dwc/terms/phylum
preparations	A list of preparations or preservation methods for specimens. http://rs.tdwg.org/dwc/terms/preparations
recordedBy	A list (concatenated and separated) of names of people, groups or organisations responsible for recording the original occurrence record. The primary collector or observer is listed first. https://rs.tdwg.org/dwc/terms/recordedBy
recordedByID	A list (concatenated and separated) of the globally unique identifier for the person, people, groups or organisations responsible for recording the original occurrence record. http://rs.tdwg.org/dwc/terms/recordedByID
scientificName	The full scientific name, with authorship and date information if known. When forming part of a specimen identification, the name is lowest level taxonomic rank that can be determined. http://rs.tdwg.org/dwc/terms/scientificName
scientificNameAuthorship	The authorship information for the scientificName. http://rs.tdwg.org/dwc/terms/scientificNameAuthorship
sex	The sex of the biological individual(s) represented in the occurrence record. http://rs.tdwg.org/dwc/terms/sex
MID_specialCollection	Internal dataset value indicating occurrence records originating from specific sources.
specificEpithet	The name of the species epithet of the scientificName. http://rs.tdwg.org/terms/specificEpithet
startDayOfYear	The earliest integer day of the year on which the collecting event occurred. http://rs.tdwg.org/terms/startDayOfYear
stateProvince	The name of the next smaller administrative region than country (state, province, canton, department, region etc.) in which the collecting event location occurs. http://rs.tdwg.org/dwc/terms/stateProvince
subgenus	The full scientific name of the subgenus in which the taxon is classified. http://rs.tdwg.org/dwc/terms/subgenus
superFamily	The full scientific name of the superfamily in which the taxon is classified. http://rs.tdwg.org/dwc/terms/superfamily
taxonID	An identifier originating from GBIF or Plecoptera Species File for a dwc:Taxon. http://rs.tdwg.org/dwc/terms/taxonID
taxonomicComments	Internal dataset column containing notes and comments about the taxon recorded.
taxonomicStatus	The status of the use of the scientificName as a label for a taxon. http://rs.tdwg.org/dwc/terms/taxonomicStatus
taxonRank	The taxonomic rank of the most specific name in the scientificName. http://rs.tdwg.org/dwc/terms/taxonRank
typeName	A scientific name that is based on a type specimen. http://rs.tdwg.org/dwc/terms/typifiedName
typeStatus	A list (concatenated and separated) of nomenclatural types (type status, typified scientific name, publication) applied to the subject. http://rs.tdwg.org/dwc/terms/typeStatus
previousIdentifications	A list (concatenated and separated) of previous assignments of names to the dwc:Organism. http://rs.tdwg.org/dwc/terms/previousIdentifications
verbatimIdentification	A string representing the taxonomic identification as it appears in the original record. http://rs.tdwg.org/dwc/terms/verbatimIdentification

## Supplementary Material

78C074CB-C43E-5C1C-BD96-8658660B75D3 10.3897/BDJ.14.e208358.suppl1 Supplementary material 1 Species occurrence data Data type occurrences Brief description Species occurrence data for stoneflies from the Midwest, USA. This file has been compressed to reduce download size. File: oo_1716431.zip https://binary.pensoft.net/file/1716431 Phillip N. Hogan, Ralph E. DeWalt, Scott A. Grubbs, Matthew Yoder

## Figures and Tables

**Table 1.** Published literature summarising most of each state's stonefly fauna known at that time. References are arranged chronologically per each state.

**State**	**Citation**	**Comments**
**Illinois**	Walsh (1862)	Described several new species
	Frison (1929), Frison (1935), Frison (1937), Frison (1942)	Statewide and regional treatments, new species described
	Webb and Harris (1993)	Technical report on rare species
	Harris and Webb (1994)	Updated nomenclature
	Webb and DeWalt (1997)	Technical report on search for *Alloperla roberti*
	DeWalt et al. (1999), DeWalt et al. (2001), DeWalt et al. (2002)	Described new species, treated small perlids
	DeWalt et al. (2005), DeWalt and Grubbs (2011)	Historical comparison, updated fauna, losses and rarity
	Webb (2002a), Webb (2002b)	Winter stonefly revisitation, Rock River stoneflies
	Cao et al. (2013)	Predicted distributions
**Indiana**	Ricker (1945)	Statewide survey
	Ricker (1952)	Described new species
	Finni (1973a)	*Allocapnia* biology, abstract only
	Finni (1973b)	Life history of winter stoneflies
	Bednarik and McCafferty (1977)	Statewide survey
	Grubbs (2004), DeWalt and Grubbs (2011)	Updated fauna
	DeWalt et al. (2016)	Survey of Indiana Dunes National Lakeshore
	Newman et al. (2021)	HUC-8 diversity analysis
**Iowa**	Heimdal et al. (2004)	Statewide survey
	Heimdal and Birmingham (2006)	Updated fauna
**Michigan**	Yanoviak and McCafferty (1996)	Ecological study of Huron Mountains
	Grubbs and Bright (2001)	Statewide survey
	Grubbs et al. (2012)	Updated fauna
	DeWalt and South (2015)	Survey of Isle Royale National Park
**Minnesota**	Harden (1942)	Association of immatures with adults
	Harden and Mickel (1952)	Statewide survey and description of new species
	Lager et al. (1979)	Survey of north-western Minnesota
**Ohio**	Say (1823)	Described first Midwest species
	Walker (1947)	Regional survey
	Gaufin (1956)	Statewide survey
	Tkac (1978)	Local survey
	Tkac (1979)	Regional surveys
	Robertson (1979), Robertson (1984)	Local Surveys
	Beckett (1987)	Ohio River surveys
	Fishbeck (1987)	Local surveys
	Bolton (2010)	Additional species record
	DeWalt et al. (2012), DeWalt et al. (2016)	Statewide survey and diversity assessments
	Grubbs et al. (2013a)	Rare species assessment
	DeWalt and Snyder (2017)	Local survey
	Bolton et al. (2019)	Added *Isoperla frisoni*
**Wisconsin**	Hilsenhoff (1970), Hilsenhoff (1975), Hilsenhoff (1995), Hilsenhoff and Narf (1972)	Keys to genera and checklists
	Hilsenhoff and Billmyer (1973)	Perlodidae of Wisconsin
	Selgeby (1974)	Lake Superior survey
	Narf and Hilsenhoff (1974)	Otter Creek survey
	Jop and Szczytko (1984)	Life cycle and production of *Isoperla signata*
	Sandberg and Szczytko 1997	Life cycle of *Isoperla lata*

**Table 2.** Summary of specimen records contributed by each institution by temporal range, total number of species, unique species (if present) and total count of occurrence records. The temporal range includes the beginning and ending years represented within each institution. Institution coden as provided by gr.bio.org (if available).

**Coden**	**Institution Name**	**Temporal Range**	**Number of Species**	**Unique Species**	**Total Records**
ANSP	Academy of Natural Sciences, Drexel University, Philadelphia, Pennsylvania	1901—1938	13	0	44
BPSC	Bill P. Stark Collection, Clinton, Mississippi	1953—2005	7	0	13
BYU	Brigham Young University, Provo, Utah	1915—2004	94	*Acroneuria hitchcocki* Kondratieff & Kirchner, 1988	2,045
CHNP	Crane Hollow Nature Preserve Collection, Rockbridge, Ohio (INHS Catalogue numbers)	1957—2015	40	0	364
CLEV	Cleveland Museum of Natural History, Cleveland, Ohio	1923—2005	22	0	72
CNC	Canadian National Collection, Ottawa, Ontario	1898—1971	87	0	724
CNHM	Cincinnati Natural History Museum, Cincinnati, Ohio	1899—1938	2	0	3
CSUIC	C.P. Gillette Museum of Arthropod Diversity, Fort Collins, Colorado	1960—2013	24	*Isoperla gibbsae* Harper, 1971, *Isoperla kirchneri* Szczytko & Kondratieff, 2015	104
DEBU	University of Guelph Insect Collection, Ontario	1983—1991	1	0	2
EIUC	Eastern Illinois University, Charleston, Illinois	2001—2002	6	0	11
FMNH	Field Museum of Natural History, Chicago, Illinois	1900—1994	30	0	273
INHS	Illinois Natural History Museum, Champaign, Illinois	1860—2025	147	*Alloperla roberti* Surdick, 1981, *Helopicus subvarians* (Banks, 1920), *Isoperla conspicua* Frison, 1935, *Isoperla namata* Frison, 1942, *Isoperla orata* Frison, 1942, *Nemocapnia carolina* Banks, 1938, *Perlesta cinctipes* (Banks, 1905)	23,558
ISIC	Iowa State University Insect Collection, Ames, Iowa	1885—2018	42	0	726
MSUARC	Michigan State University Arthropod Collection, East Lansing, Michigan	1880—2011	43	0	469
NDUC	Notre Dame University Insect Collection, South Bend, Indiana	1958—2004	19	0	218
OBS-INHS	Ohio Biological Survey, now owned by INHS	1985—2001	41	0	730
OEPA	Ohio Environmental Protection Agency, Columbus, Ohio	1973—2013	36	0	1,748
OHC	Ohio History Connection, Columbus, Ohio	1891—1975	7	0	17
OSUC	C.A. Triplehorn Insect Collection, Ohio State University, Columbus, Ohio	1880—1999	34	0	519
PERC	Purdue University Entomological Research Collection, West Lafayette, Indiana	1879—2005	73	0	2,194
PNHC	Phillip N. Hogan Collection	2021—2024	60	0	528
RKFC-INHS	Ralph F. Kirchner Collection, Huntington, West Virginia, now owned by INHS	1936—1996	25	0	188
ROM	Royal Ontario Museum, Toronto, Ontario	1899—1984	6	0	14
SIUC-INHS	Southern Illinois University, Carbondale, Illinois, now owned by INHS	1876—2002	25	0	279
SWSC-INHS	Stanley W. Szczytko Collection, now owned by INHS	1885—2011	53	0	1,383
UIHygenics	University of Iowa State Hygenic Laboratory, Iowa City, Iowa	1994—2017	14	0	559
UMMZ	University of Michigan Museum of Zoology, Ann Arbor, Michigan	1883—2009	42	0	334
UMSP	University of Minnesota St. Paul, St. Paul, Minnesota	1889—2012	62	*Isoperla emmarginata* Harden & Mickel, 1952, *Isoperla maxana* Harden & Mickel, 1952	1,970
USNM	United States National Museum, Washington D.C.	1976—1976	17	0	30
UWIRC	University of Wisconsin Insect Research Collection	1907—2003	56	0	6,284
WKUC	Western Kentucky University Collection, Bowling Green, Kentucky	1989—2022	99	*Leuctra truncata* (Claassen, 1923)	1,576
Literature		1891—2015	116	*Podmosta macdunnoughi* (Ricker, 1947)	2,045
**Total**					**49,047**

**Table 3.** A tally of 157 Plecoptera species recovered from seven Midwest, USA states. State abbreviations are ISO 3166-2 standard alpha codes. 0 = not yet found in state, 1 = present historically or contemporarily, 2 = presumed extirpated and 3 = presumed extinct. Prevalence is the total number of states with positive records for the species. Scientific names follow usage in Plecoptera Species File (DeWalt et al. 2026). Phylogenetic organisation of families follows Zwick (2000). Species reported as new state records are indicated by font in bold and the state is indicated by an asterisk (*). Species presumed extinct are indicated by a dagger (†). States are organised in southern (Ohio to Iowa) and northern (Michigan to Minnesota) tiers, both of which are ordered east to west.

**Family**	**Scientific Name**	**OH**	**IN**	**IL**	**IA**	**MI**	**WI**	**MN**	**Prevalence**
Capniidae	*Allocapnia forbesi* Frison, 1929	1	1	1	0	0	0	0	3
Capniidae	*Allocapnia frisoni* Ross & Ricker, 1964	1	0	0	0	0	0	0	2
Capniidae	*Allocapnia granulata* (Claassen, 1924)	1	1	1	1	1	1	1	7
Capniidae	*Allocapnia illinoensis* Frison, 1935	1	1	2	0	0	1	1	5
Capniidae	*Allocapnia indianae* Ricker, 1952	1	1	0	0	0	0	0	2
Capniidae	*Allocapnia minima* (Barnston, 1848)	0	0	0	0	1	1	1	3
Capniidae	*Allocapnia mystica* Frison, 1929	1	1	1	0	0	0	0	3
Capniidae	*Allocapnia nivicola* (Fitch, 1847)	1	1	1	0	0	1	0	4
Capniidae	*Allocapnia ohioensis* Ross & Ricker, 1964	1	1	0	0	0	0	0	2
Capniidae	*Allocapnia pechumani* Ross & Ricker, 1964	1	0	0	0	0	0	0	1
Capniidae	*Allocapnia pygmaea* (Burmeister, 1839)	1	0	0	1	1	1	1	5
Capniidae	*Allocapnia recta* (Claassen, 1924)	1	1	1	0	0	0	0	3
Capniidae	*Allocapnia rickeri* Frison, 1942	1	1	1	1	0	1	1	6
Capniidae	*Allocapnia smithi* Ross & Ricker, 1971	2	1	1	0	0	0	0	3
Capniidae	*Allocapnia vivipara* (Claassen, 1924)	1	1	1	1	1	1	1	7
Capniidae	*Allocapnia zola* Ricker, 1952	1	0	0	0	0	0	0	1
Capniidae	*Capnia vernalis* Newport, 1848	0	0	0	0	1	1	1	3
Capniidae	*Capnura manitoba* (Claassen, 1924)	0	0	0	0	1	0	1	2
Capniidae	*Nemocapnia carolina* Banks, 1938	0	2	2	0	0	0	0	2
** Capniidae **	***Paracapnia angulata* Hanson, 1961**	1	1*	2	0	1	1	1	6
Capniidae	*Paracapnia opis* (Newman, 1839)	0	0	0	0	1	1	1	3
Leuctridae	*Leuctra alexanderi* Hanson, 1941	1	0	0	0	0	0	0	1
Leuctridae	*Leuctra alta* James, 1974	0	1	1	0	0	0	0	2
Leuctridae	*Leuctra duplicata* Claassen, 1923	1	0	0	0	0	0	0	1
Leuctridae	*Leuctra ferruginea* (Walker, 1852)	1	0	1	0	1	1	1	5
** Leuctridae **	***Leuctra rickeri* James, 1976**	1	1	1	1	1	1*	0	6
Leuctridae	*Leuctra sibleyi* Claassen, 1923	1	1	1	0	0	1	0	4
Leuctridae	*Leuctra tenella* Provancher, 1878	1	0	0	0	0	0	0	1
Leuctridae	*Leuctra tenuis* (Pictet, 1841)	1	1	1	1	1	1	1	7
** Leuctridae **	***Leuctra truncata* Claassen, 1923**	1*	0	0	0	0	0	0	1
Leuctridae	*Paraleuctra sara* (Claassen, 1937)	1	1	0	0	0	0	0	2
Leuctridae	*Zealeuctra claasseni* (Frison, 1929)	1	1	1	0	0	0	0	3
Leuctridae	*Zealeuctra fraxina* Ricker & Ross, 1969	1	1	1	0	0	0	0	3
Leuctridae	*Zealeuctra narfi* Ricker & Ross, 1969	0	0	1	0	0	1	0	2
Nemouridae	*Amphinemura delosa* (Ricker, 1952)	1	1	1	1	1	1	1	7
Nemouridae	*Amphinemura nigritta* (Provancher, 1876)	1	0	0	0	1	0	0	2
Nemouridae	*Amphinemura palmeni* (Koponen, 1917)	0	0	0	1	1	1	1	4
** Nemouridae **	***Amphinemura varshava* (Ricker, 1952)**	1	1	1	1	1*	1	1*	7
Nemouridae	*Nemoura arctica* Esben-Petersen, 1910	1	0	1	1	1	1	1	6
Nemouridae	*Ostrocerca albidipennis* (Walker, 1852)	1	0	0	0	1	0	0	2
Nemouridae	*Ostrocerca truncata* (Claassen, 1923)	1	1	1	0	0	0	0	2
** Nemouridae **	***Paranemoura perfecta* (Walker, 1852)**	0	0	0	0	1	0	1*	2
Nemouridae	*Podmosta macdunnoughi* (Ricker, 1947)	0	0	0	0	0	0	1	1
Nemouridae	*Prostoia completa* (Walker, 1852)	1	1	1	1	1	1	1	7
Nemouridae	*Prostoia hallasi* Kondratieff & Kirchner, 1984	0	0	1	0	0	0	0	1
Nemouridae	*Prostoia ozarkensis* Baumann & Grubbs, 2014	0	0	1	0	0	0	0	1
Nemouridae	*Prostoia similis* (Hagen, 1861)	1	1	1	0	1	1	1	6
Nemouridae	*Shipsa rotunda* (Claassen, 1923)	0	0	1	1	1	1	1	5
Nemouridae	*Soyedina vallicularia* (Wu, 1923)	1	1	1	1	1	0	0	5
Taeniopterygidae	*Oemopteryx glacialis* (Barnston, 1848)	0	0	0	0	1	1	1	3
Taeniopterygidae	*Strophopteryx fasciata* (Burmeister, 1839)	1	1	1	1	1	1	1	7
Taeniopterygidae	*Taeniopteryx burksi* Ricker & Ross, 1968	1	1	1	1	1	1	1	7
Taeniopterygidae	*Taeniopteryx lita* Frison, 1942	1	1	1	0	0	0	0	3
Taeniopterygidae	*Taeniopteryx maura* (Pictet, 1841)	1	1	0	0	0	0	0	2
Taeniopterygidae	*Taeniopteryx metequi* Ricker & Ross, 1968	1	1	1	0	0	0	0	3
Taeniopterygidae	*Taeniopteryx nivalis* Fitch, 1847	1	1	1	1	1	1	1	7
Taeniopterygidae	*Taeniopteryx parvula* Banks, 1918	1	2	1	0	1	1	1	6
Peltoperlidae	*Peltoperla arcuata* Needham, 1905	1	0	0	0	0	0	0	1
Pteronarcyidae	*Pteronarcys biloba* Newman, 1838	1	0	0	0	0	0	0	1
Pteronarcyidae	*Pteronarcys dorsata* (Say, 1823)	1	1	0	0	1	1	1	5
** Pteronarcyidae **	***Pteronarcys pictetii* Hagen, 1873**	1*	1	1	1	1	1	1	7
** Chloroperlidae **	***Alloperla atlantica* Baumann, 1974**	0	0	0	0	1	1*	1	3
Chloroperlidae	*Alloperla banksi* Frison, 1942	0	0	0	0	2?	0	0	1
Chloroperlidae	*Alloperla caudata* Frison, 1934	1	1	1	0	0	0	0	3
Chloroperlidae	*Alloperla chloris* Frison, 1934	1	0	0	0	0	0	0	1
** Chloroperlidae **	***Alloperla hamata* Surdick, 1981**	0	1	1*	0	0	0	0	2
Chloroperlidae	*Alloperla idei* (Ricker, 1935)	1	0	0	0	0	0	0	1
Chloroperlidae	*Alloperla imbecilla* (Say, 1823)	1	0	0	0	0	0	0	1
Chloroperlidae	*Alloperla leonarda* Ricker, 1952	0	0	0	0	1	0	1	2
Chloroperlidae	*Alloperla petasata* Surdick, 2004	1	0	0	0	0	0	0	1
Chloroperlidae	†*Alloperla roberti* Surdick 1981	0	0	3	0	0	0	0	3
Chloroperlidae	*Alloperla usa* Ricker, 1952	1	0	0	0	0	0	0	1
Chloroperlidae	*Haploperla brevis* (Banks, 1895)	1	1	1	0	1	1	1	6
Chloroperlidae	*Haploperla orpha* (Frison, 1937)	0	0	0	0	2?	1	1	3
Chloroperlidae	*Sweltsa hoffmani* Kondratieff & Kirchner, 2009	1	1	0	0	0	0	0	2
Chloroperlidae	*Sweltsa lateralis* (Banks, 1911)	1	0	0	0	0	0	0	1
Perlidae	*Acroneuria abnormis* (Newman, 1838)	1	1	1	1	1	1	1	7
Perlidae	*Acroneuria carolinensis* (Banks, 1905)	1	0	0	0	0	0	0	1
Perlidae	*Acroneuria covelli* Grubbs & Stark, 2004	2	1	0	0	0	0	0	2
Perlidae	*Acroneuria evoluta* Klapálek, 1909	2	1	1	1	0	0	0	4
Perlidae	*Acroneuria filicis* Frison, 1942	1	2	1	0	0	0	0	3
Perlidae	*Acroneuria frisoni* Stark & Brown, 1991	1	1	1	0	1	1	0	5
Perlidae	*Acroneuria hitchcocki* Kondratieff & Kirchner, 1988	0	1	0	0	0	0	0	1
Perlidae	*Acroneuria internata* (Walker, 1852)	1	1	2	0	1	1	1	6
Perlidae	*Acroneuria kosztarabi* Kondratieff & Kirchner, 1993	1	0	0	0	0	0	0	1
** Perlidae **	***Acroneuria lycorias* (Newman, 1839)**	1	1*	0	1	1	1	1	6
Perlidae	*Acroneuria perplexa* Frison, 1937	2	1	2	0	0	0	0	3
Perlidae	*Agnetina annulipes* (Hagen, 1861)	2	1	0	0	0	0	0	2
Perlidae	*Agnetina capitata* (Pictet, 1841)	1	1	2	1	1	1	1	7
Perlidae	*Agnetina flavescens* (Walsh, 1862)	1	1	2	0	1	1	1	6
Perlidae	*Attaneuria ruralis* (Hagen, 1861)	2	2	1	1	2	1	1	7
Perlidae	*Eccoptura xanthenes* (Newman, 1838)	1	0	0	0	0	0	0	1
Perlidae	*Neoperla catharae* Stark & Baumann, 1978	1	1	1	0	1	0	0	4
Perlidae	*Neoperla clymene* (Newman, 1839)	1	1	1	1	1	1	1	7
Perlidae	*Neoperla coosa* Smith & Stark, 1998	1	1	0	0	0	1	0	3
Perlidae	*Neoperla gaufini* Stark & Baumann, 1978	1	1	0	0	0	0	0	2
Perlidae	*Neoperla harpi* Ernst & Stewart, 1986	0	0	1	0	0	0	0	1
Perlidae	*Neoperla mainensis* Banks, 1948	2	0	2	0	0	0	0	2
Perlidae	*Neoperla occipitalis* (Pictet, 1841)	1	2	2	0	1	1	0	5
Perlidae	*Neoperla osage* Stark & Lentz, 1988	0	1	0	1	0	0	0	2
Perlidae	*Neoperla robisoni* Poulton & Stewart, 1986	1	1	2	0	0	1	1	5
Perlidae	*Neoperla stewarti* Stark & Baumann, 1978	1	1	2	0	1	1	1	6
Perlidae	*Paragnetina kansensis* (Banks, 1905)	0	2	2	0	0	0	0	2
Perlidae	*Paragnetina media* (Walker, 1852)	1	1	2	1	1	1	1	7
Perlidae	*Perlesta adena* Stark, 1989	1	1	0	0	0	0	0	2
Perlidae	*Perlesta armitagei* Grubbs & DeWalt, 2018	1	1	0	0	0	0	0	2
Perlidae	*Perlesta cinctipes* (Banks, 1905)	0	0	1	1	0	0	0	2
** Perlidae **	***Perlesta decipiens* (Walsh, 1862)**	1	1	1	1	1	1	1*	7
** Perlidae **	***Perlesta ephelida* Grubbs & DeWalt, 2012**	1	1	1	1	1	1	1*	7
** Perlidae **	***Perlesta golconda* DeWalt & Stark, 1998**	0	1	1	1	2	1*	1*	6
** Perlidae **	***Perlesta lagoi* Stark, 1989**	1	1	1	1	1	1*	1*	7
** Perlidae **	***Perlesta nitida***	1*	1	0	0	1*	1*	0	4
** Perlidae **	***Perlesta ouabache* Grubbs & DeWalt, 2011**	0	1	1*	1*	0	1*	1*	5
Perlidae	*Perlesta shawnee* Grubbs, 2005	0	1	1	0	0	0	0	2
Perlidae	*Perlesta teaysia* Kirchner & Kondratieff, 1997	1	1	1	0	0	0	0	3
** Perlidae **	***Perlesta* WI-1**	0	0	0	0	1	1*	1*	3
Perlidae	*Perlesta xube* Stark & Rhodes, 1997	1	1	1	1	0	0	0	4
Perlidae	*Perlinella drymo* (Newman, 1839)	1	1	1	1	1	1	1	7
Perlidae	*Perlinella ephyre* (Newman, 1839)	1	1	1	1	1	1	1	7
Perlodidae	*Arcynopteryx dichroa* (McLachlan, 1872)	0	0	0	0	1	0	0	1
** Perlodidae **	***Clioperla clio* (Newman, 1839)**	1	1	1	1	1	1	1*	7
Perlodidae	*Cultus decisus decisus* (Walker, 1852)	1	0	0	0	1	0	0	2
Perlodidae	*Diploperla robusta* Stark & Gaufin, 1974	1	1	1	0	0	0	0	3
Perlodidae	*Helopicus nalatus* (Frison, 1942)	0	2	1	0	1	0	0	3
** Perlodidae **	***Helopicus subvarians* (Banks, 1920)**	0	0	0	0	0	0	1*	1
Perlodidae	*Hydroperla crosbyi* (Needham & Claassen, 1925)	0	1	1	1	0	0	0	3
Perlodidae	*Hydroperla fugitans* (Needham & Claassen, 1925)	0	1	1	1	0	0	0	3
** Perlodidae **	***Isogenoides doratus* (Frison, 1942)**	0	0	0	1	1	1*	0	3
Perlodidae	*Isogenoides frontalis* (Newman, 1838)	0	0	0	0	1	1	1	3
Perlodidae	*Isogenoides olivaceus* (Walker, 1852)	0	0	0	0	1	1	0	2
Perlodidae	*Isogenoides varians* (Walsh, 1862)	0	1	1	1	0	0	0	3
Perlodidae	*Isoperla bilineata* (Say, 1823)	1	1	1	1	1	1	1	7
Perlodidae	*Isoperla burksi* Frison, 1942	1	1	1	0	0	0	0	3
Perlodidae	*†Isoperla conspicua* Frison, 1935	0	0	3	0	0	0	0	1
Perlodidae	*Isoperla cotta* Ricker, 1952	0	0	0	0	1	1	1	3
Perlodidae	*Isoperla decepta* Frison, 1935	1	1	1	0	0	0	0	3
Perlodidae	*Isoperla dicala* Frison, 1942	1	1	0	1	1	1	1	6
Perlodidae	†*Isoperla emarginata* Harden & Mickel, 1952	0	0	0	0	0	0	3	1
Perlodidae	*Isoperla frisoni* Illies, 1966	1	1	0	0	1	1	1	5
** Perlodidae **	***Isoperla gibbsae* Harper, 1971**	1*	0	0	0	0	0	0	1
Perlodidae	*Isoperla holochlora* Klapálek, 1923	1	0	0	0	0	0	0	1
Perlodidae	*Isoperla irregularis* (Klapálek, 1923)	0	0	1	0	0	0	0	1
** Perlodidae **	***Isoperla kirchneri* Szczytko & Kondratieff, 2015**	1*	0	0	0	0	0	0	1
Perlodidae	*Isoperla lata* Frison, 1942	0	0	0	0	1	1	1	3
Perlodidae	*Isoperla longiseta* Banks, 1906	0	0	2	2	0	0	2	3
Perlodidae	*Isoperla marlynia* Needham & Claassen, 1925	0	2	1	1	1	1	1	6
Perlodidae	*†Isoperla maxana* Harden & Mickel, 1952	0	0	0	0	0	0	3	1
Perlodidae	*Isoperla montana* (Banks, 1898)	1	1	0	0	0	0	0	2
Perlodidae	*Isoperla namata* Frison, 1942	0	2	2	0	0	0	0	2
Perlodidae	*Isoperla nana* (Walsh, 1862)	1	1	1	0	1	1	0	5
Perlodidae	*Isoperla orata* Frison 1942	1	0	0	0	0	0	0	1
Perlodidae	*Isoperla richardsoni* Frison, 1935	1	2	2	1	1	1	1	7
Perlodidae	*Isoperla signata* (Banks, 1902)	1	0	0	1	1	1	1	5
Perlodidae	*Isoperla slossonae* (Banks, 1911)	0	0	0	1	1	1	1	4
Perlodidae	*Isoperla transmarina* (Newman, 1838)	1	0	0	1	1	1	1	5
** Perlodidae **	***Malirekus hastatus* (Banks, 1920)**	1*	0	0	0	0	0	0	1
Perlodidae	*Malirekus iroquois* Stark & Szczytko, 1988	1	0	0	0	0	0	0	1
	**Total species recovered**	109	93	85	47	71	68	65	157
	**State extirpated or extinct species**	7	9	18	1	5	0	3	
	**New state records**	5	2	2	1	1	7	9	
	**Extant species**	100	83	66	46	66	68	66	
		**OH**	**IN**	**IL**	**IA**	**MI**	**WI**	**MN**	**Prevalence**

**Table 4.** Stonefly species whose presence in the Midwest, USA is based upon historical records (pre-1980) only.

**Family**	**Species**	**Last Recorded Year**	**State(s) of Last Record**	**Total Number of Records**
Capniidae	*Nemocapnia carolina* Banks, 1938	1940	Indiana	5
Chloroperlidae	*Alloperla banksi* Frison, 1942	1947	Michigan	4
Chloroperlidae	*Alloperla roberti* Surdick, 1981	1860	Illinois	1
Chloroperlidae	*Haploperla orpha* (Frison, 1937)	1970	Minnesota	62
Nemouridae	*Podmosta macdunnoughi* (Ricker, 1947)	1979	Minnesota	1
Perlodidae	*Isoperla conspicua* Frison, 1935	1931	Illinois	1
Perlodidae	*Isoperla kirchneri* Szczytko & Kondratieff, 2015	1976	Ohio	2
Perlodidae	*Isoperla longiseta* Banks, 1906	1973	Iowa	43
Perlodidae	*Isoperla emarginata* Harden & Mickel, 1952	1939	Minnesota	1
Perlodidae	*Isoperla maxana* Harden & Mickel, 1952	1948	Minnesota	1
Perlodidae	*Isoperla namata* Frison, 1942	1950	Illinois	7
Perlidae	*Neoperla mainensis* Banks, 1948	1927	Illinois	46
Perlidae	*Paragnetina kansensis* (Banks, 1905)	1944	Illinois, Indiana	50

## References

[B13917855] Mapper Acme Acme Mapper 2.2. http://mapper.acme.com.

[B13917915] Beckett DC (1987). Plecoptera of the Ohio River: community, composition, and species phenologies of nymphs collected near Cincinnati, Ohio. Ohio Journal of Science.

[B13917940] Bednarik AF, McCafferty William P. (1977). A checklist of the stoneflies, or Plecoptera, of Indiana. Great Lakes Entomologist.

[B13917949] Bolton MJ (2010). New state records of aquatic insects for Ohio, U.S.A.. Entomological News.

[B13917958] Bolton MJ, Macy SK, DeWalt Ralph Edward, Jacobus Luke M (2019). New Ohio and Indiana records of aquatic insects (Ephemeroptera, Plecoptera, Trichoptera, Coleoptera: Elmidae, Diptera: Chironomidae). Ohio Biological Survey Notes.

[B13917976] Cao Yong, DeWalt R. Edward, Robinson Jason L., Tweddale Tari, Hinz Leon, Pessino Massimo (2013). Using Maxent to model the historic distributions of stonefly species in Illinois streams: The effects of regularization and threshold selections. Ecological Modelling.

[B14058651] DeWalt RE, Webb DW, Harris MA (1999). Summer Ephemeroptera, Plecoptera, and Trichoptera (EPT) species richness and community structure in the lower Illinois River basin of Illinois. Great Lakes Entomologist.

[B14059025] DeWalt RE, Webb DW, Kompare TN (2001). The *Perlesta
placida* (Hagen) complex (Plecoptera: Perlidae) in Illinois, new state records, distributions, and an identification key. Proceedings of the Entomological Society of Washington.

[B13918068] DeWalt RE, Webb DW, Soli AM (2002). The *Neoperla
clymene* (Newman) complex (Plecoptera: Perlidae) in Illinois, new state records, distributions, and an identification key.. Proceedings of the Entomological Society of Washington.

[B13918077] DeWalt R., South Eric J. (2015). Ephemeroptera, Plecoptera, and Trichoptera on Isle Royale National Park, USA, compared to mainland species pool and size distribution. ZooKeys.

[B14059083] DeWalt R., South Eric J., Robertson Desiree R., Marburger Joy E., Smith Wendy W., Brinson Victoria (2016). Mayflies, stoneflies, and caddisflies of streams and marshes of Indiana Dunes National Lakeshore, USA. ZooKeys.

[B13918110] DeWalt RE, Snyder ED (2017). Plecoptera of Crane Hollow Preserve, Ohio, comparison to similar efforts. Illiesia.

[B13918119] DeWalt R., Yoder Matthew, Snyder Elise, Dmitriev Dmitry, Ower Geoffrey (2018). Wet collections accession: a workflow based on a large stonefly (Insecta, Plecoptera) donation. Biodiversity Data Journal.

[B13917542] DeWalt Ralph Edward, Favret Colin, Webb Donald W. (2005). Just how imperiled are aquatic insects? A case study of stoneflies (Plecoptera) in Illinois. Annals of the Entomological Society of America.

[B13917509] DeWalt Ralph Edward, Grubbs Scott A. (2011). Updates to the stonefly fauna of Illinois and Indiana. Illiesia.

[B13917359] DeWalt Ralph Edward, Cao Yong, Tweddale Tari, Grubbs Scott A., Hinz Leon, Pessino Massimo, Robinson Jason L. (2012). Ohio USA stoneflies (Insecta, Plecoptera): species richness estimation, distribution of functional niche traits, drainage affiliations, and relationships to other states. ZooKeys.

[B13917498] DeWalt R. Edward, Grubbs Scott A., Armitage Brian J., Baumann Richard W., Clark Shawn M., Bolton Michael J. (2016). Atlas of Ohio aquatic insects: Volume II, Plecoptera. Biodiversity Data Journal.

[B13917446] DeWalt Ralph Edward, Ower Geoffrey Donald (2019). Ecosystem services, global diversity, and rate of stonefly species descriptions (Insecta: Plecoptera). Insects.

[B13917408] DeWalt Ralph Edward, Neu-Becker U, Stueber G Plecoptera Species File. https://plecoptera.speciesfile.org/.

[B14068722] Eichert Anna, de Almeida Lucas Henrique, Du Yu-Zhou, Duarte Tácio, Fochetti Romolo, Hotaling Scott, Huo Qing-Bo, Jouault Corentin, Kirkaldy Abigail Puleng, Letsch Harald, Li Weihai, López-Rodríguez Manuel Jesús, Machingura James, McCulloch Graham, Mo Raorao, Mtow Shodo, Pessacq Pablo, Rippel Mellis Layra Soares, Rivera-Pomar Rolando, Sproul John S, Sarmento Felipe Ribeiro Pereira, Sroka Pavel, Tierno de Figueroa José Manuel, Ware Jessica (2025). Stonefly systematics: past, present, and future. Insect Systematics and Diversity.

[B14068751] Elith Jane, Leathwick John R. (2009). Species distribution models: Ecological explanation and prediction across space and time. Annual Review of Ecology, Evolution, and Systematics.

[B13918129] Finni GR (1973). The winter stonefly genus *Allocapnia* in Indiana (Plecoptera: Capniidae). Proceedings of the Indiana Academy of Science.

[B13918138] Finni GR (1973). Biology of winter stoneflies in a central Indiana stream (Plecoptera). Annals of the Entomological Society of America.

[B13918147] Fishbeck DW (1987). Stoneflies (Plecoptera) in Gray's Run in northeastern Ohio. Ohio Journal of Science.

[B13918165] Fochetti Romolo, Tierno de Figueroa José Manuel (2007). Global diversity of stoneflies (Plecoptera; Insecta) in freshwater. Hydrobiologia.

[B13918182] Frison Theodore H. (1929). Fall and winter stoneflies, or Plecoptera, of Illinois. Illinois Natural History Survey Bulletin.

[B13918191] Frison Theodore H. (1935). The stoneflies, or Plecoptera, of Illinois. Illinois Natural History Survey Bulletin.

[B13918744] Frison Theodore H. (1937). Studies of Nearctic Insects II. Descriptions of Plecoptera with special reference to the Illinois species. Illinois Natural History Survey Bulletin.

[B13918200] Frison Theodore H. (1942). Studies of North American Plecoptera with special reference to the fauna of Illinois. Illinois Natural History Survey Bulletin.

[B13918209] Gaufin Arden F. (1956). An annotated list of the stoneflies of Ohio. Ohio Journal of Science.

[B13918218] Gaufin Arden F., Tazewell CM (1956). Aquatic macro-invertebrate communities as indicators of organic pollution in Lytle Creek. Sewage and Industrial Wastes.

[B13918235] Gibson A (1945). Obituary Theodore Henry Frison. The Canadian Entomologist.

[B13918273] Grubbs Scott A. (2004). Studies of Indiana stoneflies (Plecoptera) with an annotated and revised state checklist. Proceedings of the Entomological Society of Washington.

[B13918291] Grubbs Scott, DeWalt R. E. (2012). *Perlesta
ephelida*, a new Nearctic stonefly species (Plecoptera, Perlidae). ZooKeys.

[B13917455] Grubbs Scott A., Pessino Massimo, DeWalt Ralph Edward (2012). Michigan Plecoptera (stoneflies): distribution patterns and an updated species list. Illiesia.

[B13918300] Grubbs Scott A., Pessimo Massimo, DeWalt R. Edward (2013). Distribution Patterns of Ohio Stoneflies, with an Emphasis on Rare and Uncommon Species. Journal of Insect Science.

[B13918309] Grubbs Scott A., Kondratieff Boris C., Stark Bill P., DeWalt Ralph E. (2013). A review of the Nearctic genus *Zealeuctra* Ricker (Plecoptera, Leuctridae), with the description of a new species from the Cumberland Plateau region of eastern North America. ZooKeys.

[B13918318] Grubbs Scott A., Baumann RW, DeWalt RE, Tweddale T (2014). A review of eastern Nearctic *Prostoia* (Ricker) (Plecoptera: Nemouridae), including the description of a new species and a s surprising range extension for *P.
hallasi* Kondratieff & Kirchner. ZooKeys.

[B13918345] Grubbs Scott A., DeWalt Ralph Edward (2018). *Perlesta
armitagei* n. sp. (Plecoptera: Perlidae): More cryptic diversity in darkly pigmented Perlesta from the eastern Nearctic. Zootaxa.

[B14430568] Grubbs Scott A., Baumann Richard W. (2023). The Nemourinae (Insecta, Nemouridae) of the eastern Nearctic. Zootaxa.

[B14430586] Grubbs Scott A., Verdone Chris J., Myers Luke W., DeWalt R. Edward (2025). New Stonefly Synonymy Changes Conservation Outlook: 100-Year-Old Specimens and Integrated Taxonomy Clarify Species Concepts and Distributions of Several Eastern Nearctic Stripetails (Perlodidae: Isoperla Banks, 1905). Diversity.

[B13918244] Grubbs Scott A, Bright E (2001). *Arcynopteryx
compacta* (Plecoptera: Perlodidae), a Holarctic stonefly confirmed from Lake Superior, with a review and first checklist of the stoneflies of Michigan. Great Lakes Entomologist.

[B13918505] Harden Philip H. (1942). The Immature Stages of Some Minnesota Plecoptera. Annals of the Entomological Society of America.

[B13917490] Harden Philip E., Mickel Clarence E. (1952). The stoneflies of Minnesota (Plecoptera). https://hdl.handle.net/11299/108234.

[B13918514] Harris Mitchell A., Webb Donald W. (1994). The stoneflies (Plecoptera) of Illinois revisited. Journal of the Kansas Entomological Society.

[B13917417] Hart Lily Veronica, DeWalt Ralph Edward, Hogan Phillip Nathaniel, Grubbs Scott A, Burton David K. (2025). The stoneflies (Plecoptera) of Arkansas: a checklist compiled from museum specimen data. Biodiversity Data Journal.

[B13918532] Heimdal DP, DeWalt RE, Wilton WF (2004). Annotated checklist of the stoneflies (Plecoptera) of Iowa. Proceedings of the Entomological Society of Washington.

[B13918523] Heimdal DP, Birmingham DW (2006). Additions and corrections to the stoneflies (Plecoptera) of Iowa. Great Lakes Entomologist.

[B13918541] Hilsenhoff WL (1970). Key to genera of Wisconsin Plecoptera (stonefly) nymphs, Ephemeroptera (mayfly) nymphs and Trichoptera (caddisfly) larvae. Wisconsin Department of Natural Resources Research Report.

[B13918550] Hilsenhoff WL, Narf RP (1972). Aquatic insects of the Pine-Popple River, Wisconsin. Plecoptera (stoneflies). Wisconsin Department of Natural Resources Technical Bulletin.

[B13918559] Hilsenhoff WL, Billmyer SJ (1973). Perlodidae (Plecoptera) of Wisconsin. Great Lakes Entomologist.

[B13918568] Hilsenhoff WL (1975). Aquatic insects of Wisconsin: generic keys and notes on biology, ecology and distribution. Wisconsin Department of Natural Resources Technical Bulletin.

[B13918577] Hilsenhoff WL (1977). Use of arthropods to evaluate water quality of streams. Wisconsin Department of Natural Resources Technical Bulletin.

[B13918586] Hilsenhoff WL (1982). Using a biotic index to evaluate water quality in streams. Wisconsin Department of Natural Resources Technical Bulletin.

[B13918595] Hilsenhoff WL (1987). An improved biotic index of organic stream pollution. Great Lakes Entomologist.

[B13918604] Hilsenhoff WL (1995). Aquatic insects of Wisconsin. Keys to Wisconsin genera and notes on biology, habitat, distribution, and species. Natural History Museums Council, University of Wisconsin-Madison.

[B13918613] Jop Krzysztof, Szczytko Stanley W. (1984). Life cycle and production of *Isoperla
signata* in a Central Wisconsin trout stream. Aquatic Insects.

[B13918658] Lager TM, Johnson MD, Williams SN, McCullough JL (1979). A preliminary report on the Plecoptera and Trichoptera of northeastern Minnesota. Great Lakes Entomologist.

[B13918667] Lenat David R. (1993). A Biotic Index for the Southeastern United States: Derivation and list of tolerance values, with criteria for assigning water-quality ratings. Journal of the North American Benthological Society.

[B14059488] Myers L, Kondratieff BC (2017). Larvae of North American species of *Pteronarcys* (Plecoptera: Pteronarcyidae). Illiesia.

[B13918676] Myers Luke, Kondratieff Boris, Grubbs Scott, Pett Lindsey, DeWalt R. Edward, Mihuc Timothy, Hart Lily (2025). Distributional and species richness patterns of the stoneflies (Insecta, Plecoptera) in New York State. Biodiversity Data Journal.

[B13918688] Narf RP, Hilsenhoff WL (1974). Emergence patterns of stoneflies (Plecoptera) in Otter Creek Wisconsin. Great Lakes Entomologist.

[B13918697] Needham JH, Claassen PW (1925). A monograph of the Plecoptera or stoneflies of America north of Mexico. Thomas Say Foundation, Entomological Society of America.

[B13917428] Newman Evan A., DeWalt Ralph Edward, Grubbs Scott A. (2021). Plecoptera (Insecta) diversity in Indiana: a watershed-based analysis. Diversity.

[B14099638] Oksanen J., Simpson G., F. Blanchet, Kindt R., Legendre P., Minchin P., O'Hara R., Solymos P., Stevens M., Szoecs E., Wagner H., Barbour M., Bedward M., Bolker B., Borcard D., Borman T., Carvalho G., Chirico M., De Caceres M., Durand S., Evangelista H., FitzJohn R., Friendly M., Furneaux B., Hannigan G., Hill M., Lahti L., Martino C., McGlinn D., Ouellette M., Ribeiro Cunha E., Smith T., Steir A., Ter Braak C., Weedon J. (2026). vegan: Community Ecology Package. https://vegandevs.github.io/vegan/.

[B14068713] Pond Gregory J. (2011). Biodiversity loss in Appalachian headwater streams (Kentucky, USA): Plecoptera and Trichoptera communities. Hydrobiologia.

[B14059094] Ricker WE (1945). A first list of Indiana stoneflies (Plecoptera). Proceedings of the Indiana Academy of Science.

[B14059103] Ricker WE (1952). Systematics studies in Plecoptera. Indiana University Publication Series.

[B14059192] Robertson DJ (1979). An annotated list of stoneflies (Plecoptera) from Penitentiary Glen, Lake County, Ohio. Great Lakes Entomologist.

[B14059201] Robertson DJ (1984). The aquatic insect community in Penitentiary Glen, a Portage Escarpment stream in northeast Ohio. Ohio Journal of Science.

[B14059134] Sandberg JB, Szczytko SW (1997). Life cycle of *Isoperla
lata* (Plecoptera: Perlodidae) in a Central Wisconsin trout stream. The Great Lakes Entomologist.

[B14058884] Say T (1823). Description of insects belonging to the order Neuroptera Linn., Latr. collected by the expedition authorized by J.C. Calhoun, Secretary of War, under the command of Major S.H. Long. The Western Quarterly Reporter of Medical, Surgical, and Natural Science.

[B14059143] Selgeby JH (1974). Immature insects (Plecoptera, Trichoptera, and Ephemeroptera) collected from deep water in western Lake Superior. Journal of the Fisheries Research Board of Canada.

[B14180083] South E. J., DeWalt R. E., Davis M. A., Thomas M. J. (2019). A new stonefly species (Plecoptera, Perlidae) from the Interior Highlands USA, with morphological and molecular comparison to other congeneric species. ZooKeys.

[B14430577] Stark Bill P., Baumann Richard. W. (1978). New species of Nearctic Neoperla (Plecoptera: Perlidae), with notes on the genus. The Great Basin Naturalist.

[B14059419] Stark BP, Stark BP, Armitage BJ (2004). The stoneflies (Plecoptera) of eastern North America.

[B13918753] Stewart KW (2002). Celebration of Bill Ricker's life and professional contributions to Plecopterology. Perla.

[B14059541] Stewart KW, Stark B P (2002). Nymphs of North American stonefly genera.

[B14059313] Szczytko SW, Kondratieff BC (2015). A review of the eastern Nearctic Isoperlinae (Plecoptera: Perlodidae) with the description of twenty-two new species. Monographs of Illiesia.

[B14059161] Tkac MA (1978). Annotated list of stoneflies (Plecoptera) from Stebbins Gulch in northeastern Ohio. Great Lakes Entomologist.

[B14059170] Tkac MA (1979). The Plecoptera of Northeastern Ohio.

[B14099591] Tucker Edna Armstrong (1929). Benjamin D. Walsh - First State Entomologist of Illinois. Transactions of the Illinois Historical Society.

[B14059771] Survey United States Geological (2019). National Hydrography Dataset. https://www.usgs.gov/core-science-systems/ngp/national-hydrography/access-national-hydrography-products.

[B14059152] Walker JD (1947). A list of the stoneflies, Plecoptera, known to occur in southeastern Ohio. Ohio Journal of Science.

[B14058921] Walsh BD (1862). List of the Pseudoneuroptera of Illinois contained in the cabinet of the writer, with descriptions of over 40 new species, and notes on their structural affinities. Proceedings of the Academy of Natural Sciences of Philadelphia.

[B14099629] Walther B. A., Morand S. (1998). Comparative performance of species richness estimation methods. Parasitology.

[B14058975] Webb DW, Harris MA (1993). Survey of 21 rare species of stoneflies (Plecoptera) in Illinois.. Illinois Natural History Survey Center for Biodiversity Technical Report.

[B14059016] Webb DW, DeWalt RE (1997). A search for *Alloperla
roberti* in northwestern Illinois (Plecoptera: Chloroperlidae). Illinois Natural History Survey Center for Biodiversity Technical Report.

[B14059056] Webb DW (2002). The winter stoneflies of Illinois (Insecta: Plecoptera): 100 years of changes. Illinois Natural History Survey Bulletin.

[B14059065] Webb DW (2002). A review of the stoneflies of the Rock River, Illinois. Illinois Natural History Survey Center for Biodiversity Technical Report.

[B14059074] Yanoviak SP, McCafferty WP (1996). Comparison of macroinvertebrate assemblages inhabiting pristine streams in the Huron Mountains of Michigan, USA. Hydrobiologia.

[B14058412] Zwick Peter (2000). Phylogenetic system and zoogeography of the Plecoptera. Annual Review of Entomology.

